# Complexity of Murine Cardiomyocyte miRNA Biogenesis, Sequence Variant Expression and Function

**DOI:** 10.1371/journal.pone.0030933

**Published:** 2012-02-03

**Authors:** David T. Humphreys, Carly J. Hynes, Hardip R. Patel, Grace H. Wei, Leah Cannon, Diane Fatkin, Catherine M. Suter, Jennifer L. Clancy, Thomas Preiss

**Affiliations:** 1 Molecular Genetics Division, Victor Chang Cardiac Research Institute, Sydney, New South Wales, Australia; 2 The John Curtin School of Medical Research, The Australian National University, Canberra Australian Capital Territory, Australia; 3 Molecular Cardiology Division, Victor Chang Cardiac Research Institute, Sydney, New South Wales, Australia; 4 St. Vincent's Clinical School, University of New South Wales, Sydney, New South Wales, Australia; 5 Cardiology Department, St Vincent's Hospital, Sydney, New South Wales, Australia; 6 School of Biotechnology and Biomolecular Sciences, University of New South Wales, Sydney, New South Wales, Australia; French National Center for Scientific Research - Institut de biologie moléculaire et cellulaire, France

## Abstract

microRNAs (miRNAs) are critical to heart development and disease. Emerging research indicates that regulated precursor processing can give rise to an unexpected diversity of miRNA variants. We subjected small RNA from murine HL-1 cardiomyocyte cells to next generation sequencing to investigate the relevance of such diversity to cardiac biology. ∼40 million tags were mapped to known miRNA hairpin sequences as deposited in miRBase version 16, calling 403 generic miRNAs as appreciably expressed. Hairpin arm bias broadly agreed with miRBase annotation, although 44 miR* were unexpectedly abundant (>20% of tags); conversely, 33 -5p/-3p annotated hairpins were asymmetrically expressed. Overall, variability was infrequent at the 5′ start but common at the 3′ end of miRNAs (5.2% and 52.3% of tags, respectively). Nevertheless, 105 miRNAs showed marked 5′ isomiR expression (>20% of tags). Among these was miR-133a, a miRNA with important cardiac functions, and we demonstrated differential mRNA targeting by two of its prevalent 5′ isomiRs. Analyses of miRNA termini and base-pairing patterns around Drosha and Dicer cleavage regions confirmed the known bias towards uridine at the 5′ most position of miRNAs, as well as supporting the thermodynamic asymmetry rule for miRNA strand selection and a role for local structural distortions in fine tuning miRNA processing. We further recorded appreciable expression of 5 novel miR*, 38 extreme variants and 8 antisense miRNAs. Analysis of genome-mapped tags revealed 147 novel candidate miRNAs. In summary, we revealed pronounced sequence diversity among cardiomyocyte miRNAs, knowledge of which will underpin future research into the mechanisms involved in miRNA biogenesis and, importantly, cardiac function, disease and therapy.

## Introduction

microRNAs (miRNAs) are small non-coding RNAs (∼19–24 nucleotides) whose regulation of mRNA translation and decay provides robustness and precision to gene expression. Precise gene regulation is crucial in the heart, where small deviations in function and structure can have devastating consequences for the organism. miRNA action is intimately entwined with signaling and transcriptional pathways to modulate cardiac development, function and disease [1], [2], [3] and a number of individual miRNAs underpin key developmental processes and cardiac diseases. For example, the MyomiRs miR-208a, -208b and -499 control myosin heavy chain isoform expression [4], miR-133a and miR-1 are crucial regulators of cardiac differentiation and development [1] and miR-195 overexpression is sufficient to induce hypertrophy in mice, while ablation of miR-208a is protective [5]. miRNA-related gene therapies for cardiac conditions are also being considered. For example, overexpression of miR-210 in the mouse model improved ventricular performance and decreased apoptosis after myocardial infarction [6], while inhibition of miR-21 reduced pathological remodeling and fibrosis in response to pressure overload [7]. It is thus important to fully understand the breadth and depth of the cardiomyocyte miRNA repertoire.

miRNAs are loaded into an Argonaute protein and guide RNA silencing complexes (RISC) to mRNAs through base pairing between the miRNA “seed” (nucleotides 2–8) and 3′ untranslated region (UTR) binding sites. Binding of RISC to the target mRNA typically inhibits translation and stimulates mRNA decay [8], [9]. miRNAs originate from genome-encoded precursors, pri-miRNAs, with characteristic hairpin structures (miRNA biogenesis reviewed in refs. [10], [11], [12], [13], [14]). The pri-miRNA is recognised and processed in the nucleus by the Microprocessor complex, which contains the endonuclease Drosha. Cleavage by Drosha ∼11 base pairs from the bottom of the hairpin yields pre-miRNA. In the cytoplasm, Dicer cleaves the pre-miRNA ∼22 nucleotides in from the Drosha cut to produce a miRNA duplex [15]. The “mature” strand of this duplex is then transferred to Argonaute, while the other strand (called passenger strand or miR*) is thought to be non-functional and commonly decayed. Strand selection is at least in part determined by the strength of base-pairing at the ends of the duplex, however relative mature miRNA/miR* expression levels can vary widely between tissues, suggesting the existence of additional regulatory mechanisms [11], [16]. Furthermore, many miR* have been observed in RISC complexes, supporting the contention that these actively repress mRNAs *in vivo* [17]. Recent reports of miRNA length and sequence variants [18], [19], [20] add further complexity to our view of what constitutes a functional miRNA. 5′ start site and 3′ end variants (called 5′ and 3′ isomiRs) may have altered targeting and/or turnover properties and can be expressed in a tissue- and development-specific manner, suggesting regulated production and biological purpose [21], [22].

Previous studies (reviewed in ref. [2]) documenting expression levels of miRNAs in the heart, and how these change in cardiac disease, mostly relied on microarray measurements, which typically cannot fully differentiate sequence variation. The field thus lacks critical information on miRNA variant expression, which could profoundly impact on our view of miRNA effects on cardiac gene expression, the definition of their mRNA targets and how we might devise experimental and therapeutic means to modulate their function. This new appreciation of miRNA complexity motivated us to generate a reference compendium of cardiomyocyte miRNA variant expression. Thus, we exhaustively sequenced the miRNA population of the murine HL-1 cell line as a pure source of functional cardiomyocytes. Our results revealed numerous examples of unexpected miRNA strand bias, sequence variation as well as novel candidate miRNAs, the existence of which will be of importance in future studies of cardiac biology and more broadly, miRNA biogenesis. We further showed that two prevalent 5′ isomiRs of miR-133a can have different targeting properties *in vivo*, directly demonstrating the biological relevance of miRNA sequence variants.

## Materials and Methods

### Cell culture and source of cardiac tissue

Ethics statement: Animals were maintained and experimental procedures were performed according to protocols approved by the Garvan and St.Vincent Hospital Animal Ethics Committee (AEC#: 09/09).

HL-1 cells were provided by W.C. Claycomb and maintained as per instructions [23]. Cardiac left ventricular tissue was taken from an inbred FVB/N mouse strain.

### Library preparation and sequencing

Total RNA was prepared from HL-1 cultures and cardiac tissue using the TRIzol® reagent. Small RNA <70 nt was enriched by flashPAGE™ electrophoresis. Libraries were created with the SOLiD™ Small RNA Expression Kit and sequenced using SOLiD™ version 2 reagents.

### Sequence analysis pipeline

We refer to an individual deep sequence read as a tag and the number of times it occurred as a count. The SOLiD™ Small RNA Pipeline was used to map tags to miRBase version 16 and the mouse genome (mm37 assembly). A schematic of our tag mapping approach is shown in Figure S1 and details of tags mapped to miRbase are located in Datasets S1, S2 and Table S1. Trimmed tags from a previously described dataset [22] were mapped to miRbase version 16, allowing one mismatch.

### miRNA target prediction and gene function analysis

Target prediction was done using Targetscan [24]. Enrichment of gene function terms was determined with IPA software (Ingenuity® Systems), using the Benjamini-Hochberg multiple testing correction method and a threshold p-value <0.01.

### Analysis of sequence and structural composition around miRNA processing sites

Nucleotide frequency at positions either side of the presumed processing sites was determined for each miRNA isomiR variant. Background nucleotide frequency was a combination of all analysed positions. For analysis of RNA structure around processing sites hairpins were visually inspected using predicted secondary structures as deposited in miRBase.

### Quantitative RT-PCR

Quantitative RT-PCR was performed as previously described and designed for annealing at 58°C [25]. Primers are listed in Table S2 and were designed against the dominant 3′ variant. For high stringency PCR to validate novel miRNA expression, annealing temperatures were increased (61°C for miR-30e-as; 62°C for 10 cycles then reducing by 0.3°C per cycle until reaching 58°C for miR-N4, -N4*, -N29 and -N29*). Melt curve analysis was performed for all experiments.

### Reporter constructs and assays

The miRNA sites were cloned into the 3′UTR of Renilla luciferase in the psiCHECK™-2 Vector (Promega). These reporters and MISSION® microRNA mimics (Sigma) were used in transient transfection assays as previously described [9].

A full description of Materials and Methods is available in Text S1.

## Results

### miRNA expression profile of HL-1 cells

**Figure 1:**
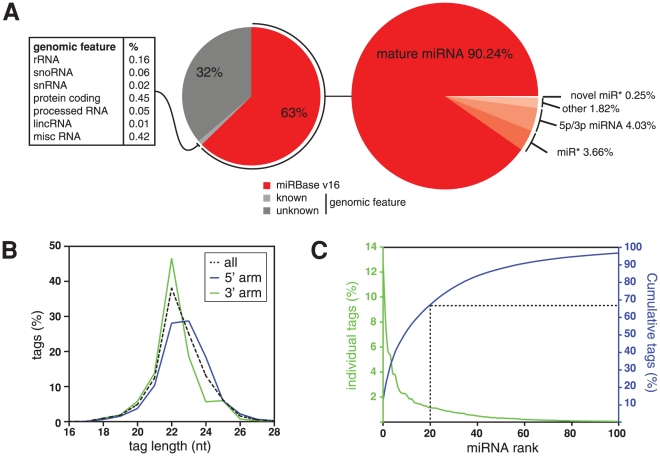
Identification of HL-1 cardiomyocyte miRNAs by next generation sequencing. (**A**) Distribution of 66,405,108 mappable small RNA sequence tags derived from HL-1 cells across miRBase version 16 and the mm37 mouse genome assembly (on left). Split of miRBase-mapped tags across different miRNA annotations (on right). (**B**) Distribution of tag lengths for all generic miRBase-mapped tags (dashed line), or separately for those derived from the 5′ or 3′ arm of precursor hairpins (blue and green lines, respectively). (**C**) Distribution of tag counts across the one hundred most abundant miRNAs. Contribution of individual miRNAs to total tag count (green), or cumulative tag contribution (blue) is plotted against ranked miRNA abundance.

HL-1 cardiomyocytes were seeded into plates at low density and cultured over a four-day period. As previously described [23], we observed a transition from non-beating cells (at day one) to more than 90% of cells beating by day four. Total RNA was extracted at 24-hour intervals, size fractionated and small RNA libraries prepared for SOLiD™ next generation sequencing. Samples were also taken from an independent repeat of the time course (except for day two), giving a total of seven libraries. Libraries were sequenced to a depth of 1.30 to 2.55×10^7^ tags (up to 35 nucleotides in length), yielding a total of 121,138,457 usable tags. To identify known miRNAs and their processing isoforms, these tags were first mapped to the 672 miRNA hairpin sequences listed in miRBase version 16 (abbreviated to miRBase in the following). During preparation and revision of this manuscript miRBase versions 17 and 18 were successively released, and thus we refer to notable differences in findings where appropriate. The remaining tags were then mapped to the mouse genome (mm37 assembly) to discover novel miRNAs (see below). Thus, using the tools and parameters detailed in the Text S1 and Figure S1, we were able to map 66,405,108 tags across the seven libraries (∼55% of usable tags; Figure 1A). Analysis of individual library data revealed that they were highly similar to each other, indicating that progression from a non-beating to a beating state did not correlate with pronounced changes in cardiomyocyte miRNA profile. Nevertheless, we noted a 52% increase in overall miRNA abundance (Text S1 and Figure S2), as seen with other cell types as they reach confluency [26]. We therefore considered these libraries as one merged set to increase the depth of coverage for our subsequent analyses.

42,451,913 tags mapped to miRBase-listed miRNA hairpins covering 1133 generic miRNA features (i.e. tags started within ±3 nt of the 5′ end of a known miRNA or were appropriately located on the opposite hairpin arm of known miRNAs, see Figure S3; Dataset S1 and Table S1 contain detailed information on miRNA sequence and tag counts). Consistent with expectation, the modal tag length was 22 nucleotides (nt; Figure 1B) and 1035 of these features were already annotated in miRBase, while the remaining 98 features potentially represent novel miR*. A recent study sequenced murine miRNA populations from adult brain, ovary, testes and several embryonic stages at a similar depth in aggregate [22]. For comparison we applied an expression threshold equivalent to theirs (≥25 tags) and detected 374 of 408 known miRNAs also observed by these investigators; we saw 113 of 177 miRNAs they did not detect, and 46 of 158 of the miRNAs discovered as novel in the previous study. We consider this a good level of overlap, given the differences in source material, library preparation methods and sequencing technology (ie. Illumina versus SOLiD™ [27]). In the following, we generally applied a more stringent expression threshold of 150 tags per miRNA to exclude miRNAs of extremely low abundance, and to focus on observations of particular relevance to cardiomyocyte biology. This retained 99.95% of mapped tags, excluded 632 miRNAs of extremely low abundance and left 403 known miRNAs confidently detected (Table S3).

The 20 most abundant miRNAs in HL-1 cells contributed 66% of all miRBase-mapped tags (Figure 1C and Table S4), with the cardiovascular miR-145 [28] alone contributing 13% of tags. Altogether, a set of 139 miRNA hairpins with established cardiac function or differential expression during heart development and disease [29], referred to as ‘cardiac miRNA set’ hereafter, gave rise to 88% of tags and 181 miRNA species (with ≥150 tags). As HL-1 cells are immortalised cardiomyocytes (though maintaining a differentiated phenotype with ability to contract and adult cardiomyocyte gene expression [30]) we wanted to cross-reference our results with an *in vivo* cardiac setting. In related work, we had sequenced a small RNA library derived from a biopsy of murine cardiac left ventrical at lower depth (3,639,611 tags mapped to miRBase). Although the biopsy contained cardiomyocytes in mixture with other cell types, its miRNA profile had clear similarities to that of the HL-1 cells (Spearman's rank correlation coefficient of 0.61; data not shown). We also noted that HL-1 cells expressed higher levels of miR-208a than miR-208b, seen as a marker of adult rather than embryonic cardiac tissue [1].

There were also differences between the heart biopsy and HL-1 datasets, the most notable being the higher miR-145 levels and lower miR-1 levels in HL-1 cells. A recent study profiling abundant miRNAs in the whole mouse heart by llumina sequencing reported miR-1 as the most abundant miRNA, contributing ∼40% of tags [31]. In the biopsy of the mouse left ventricle we also found that miR-1 was the most abundant miRNA, contributing 23% of tags. However in the atrial-derived HL-1 cell line miR-1 is the third most abundant miRNA contributing 6.3% of all miRNA reads. The 20 most abundant miRNAs from [31] contributed 47.8% of miRNA reads for HL-1 dataset and 70.8% of the miRNA reads from our cardiac dataset, suggesting that the miRNA population of the whole heart is more similar to our ventrical biopsy than HL-1 cells. Furthermore, in adult tissue there is evidence that miR-145 is expressed to a much greater extent in the atrium *versus* the ventricle [32]. As the ventricle contributes the bulk of material to the whole heart tissue as used in ref. [31], and the entire material for our heart biopsy, we would suggest that the predominance of miR-145 (and possibly the reduced miR-1 levels) in the HL-1 dataset is due to its atrial origin. In aggregate, these results indicate our detection of miRNAs in HL-1 cardiomyocytes is sensitive and consistent with expression in the adult heart.

### miRNAs with unexpected strand bias in cardiomyocytes

**Figure 2:**
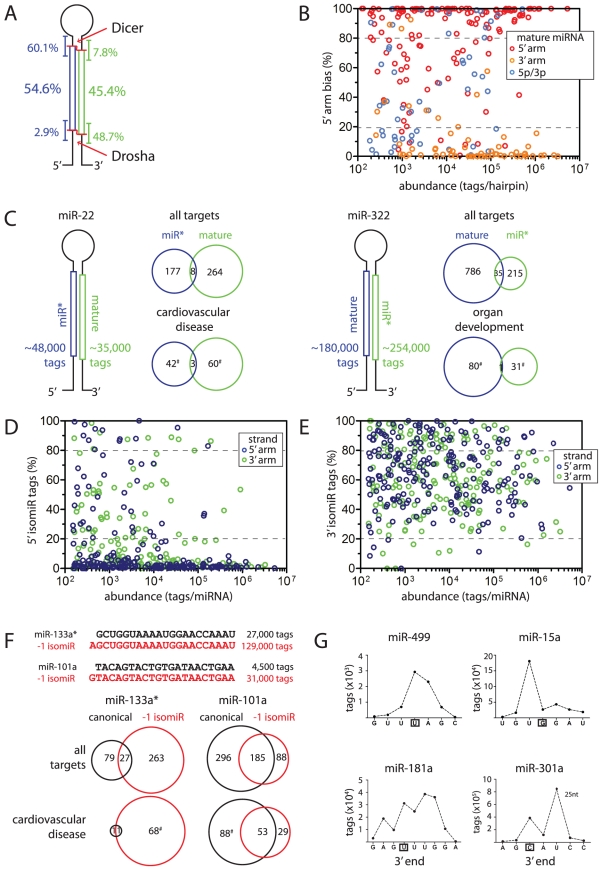
Diversity of HL-1 cardiomyocyte miRNA processing. (**A**) Schematic of hairpin miRNA precursor showing proportion of tags derived from either 5′ (blue) or 3′ (green) arm (in % of total hairpin-mapped tags; shown at center of boxed regions). For each arm, the proportion of tags representing 5′ start or 3′ end positions that vary from miRBase annotation is also shown (in % of tags per arm; shown at ends of boxed regions). Red lines indicate typical Dicer and Drosha processing sites. (**B**) 5′ strand bias of each miRNA is plotted against the sum of tags per hairpin. Color scheme represents miRBase version 16 annotation as mature species on 5′ or 3′ arms (red and orange circles, respectively), or as a 5p/-3p pair (blue circles). (**C**) Examples of miRNAs with unexpected strand bias. Schematics of hairpin indicate abundance of tag mapping to opposing arms (on left), while Venn diagrams indicate number and overlap of predicted mRNA targets (Targetscan) for each of these miRNA (on right; top: all predicted targets, bottom: only targets with specific gene function annotation (Ingenuity) as listed; #: significant enrichment of gene function term, p<0.01). (**D**) The proportion of tags representing 5′ isomiRs for each miRNA is plotted against tag abundance. Color scheme represents position of miRNA on 5′ (blue) or 3′ (green) arm. (**E**) As in (D) but showing prevalence of 3′ isomiRs. (**F**) Examples of miRNAs with high 5′ isomiR proportion. Tag sequences representing canonical miRNA (black) and major 5′ isomiR (red) are depicted on top, while analyses of mRNA targets (as in D) are shown below. (**G**) Examples of distinct miRNA 3′ end variability. Distribution of tag 3′ ends for miR-499 is from the heart biopsy, while HL-1 cell data is shown for miR-181a, miR-15a and miR-301a (miRBase-annotated 3′ end positions are boxed). Panels (B, D and E) depict only generic miRNAs (tags with 5′ start position +/− 3 nt of miRbase v16 annotation or novel miR* directly juxtaposed to a known miRNA) with an expression level of ≥150 tags; the dashed lines show tag proportion thresholds of 80% and 20% used throughout this study to categorize miRNAs.

We next determined miRNA strand bias in the HL-1 dataset and found that 90.24% of tags mapped to mature miRNAs, 3.66% to miR*, while 4.03% aligned to -5p/-3p annotated miRNAs (Figure 1A), confirming the notion that strand selection in the main is asymmetrical. The remaining 2.07% of tags mapped to non-canonical miRNA species (see below). There was a minor preference for miRNAs to be situated on the 5′ arm rather than the 3′ arm (54.6% and 45.4% of tags, respectively, Figure 2A), consistent with previous reports [33].

Despite the overall concordance with miRBase strand annotation, there were 44 mature miRNAs with abundant miR* forms. 11 cases represented a clear reversal of strand preference, with the miR* represented by >80% of tags mapped to the hairpin. Between 20–80% of tags mapped to the miR* for 33 others, thus we observed relatively symmetrical hairpin processing for these rather than the expected bias for the mature form. Conversely, 33 pairs of -5p/-3p annotated miRNAs unexpectedly displayed marked bias in their expression (>80% tags mapping to one strand). Figure 2B displays the observed strand bias for all confidently detected miRNAs, while Table 1 lists the most abundant examples for each category. Of the 77 examples with unexpected strand bias noted in HL-1 cells we could detect 51 in the lower depth cardiac biopsy dataset and saw similar strand bias for 46 (90%), confirming broad applicability of our findings to the *in vivo* cardiac setting. To assess how stable this biased strand expression was across divergent tissue types, we used our methodology to re-map and analyse miRNA sequencing data for several other murine tissues [22] and categorized miRNA expression in these tissues as for HL-1 cells in Table 1. Here we found 26 cases of unexpected miRNA strand bias in HL-1 cells that was either not seen in these other tissues or not as pronounced (Table S5). A further 6 of these were not appreciably expressed in the other tissues (<10 tags in all tissues; miR-147*, miR-463*, miR-1943*, miR-1933-3p, miR-883-3p and miR-2145-1*, the latter removed from miRbase 17) and thus their unusual strand biases have not previously been highlighted. Notable examples of highly expressed miRNAs with unexpected strand bias in HL-1 cells and also in the heart, but having very different strand bias in other tissues [22] are the abundant miR-22*, -322*, -872*, and let-7d*, as well as a marked -5p bias for miR-151-5p.

**Table 1:** miRNAs with strand reversal, symmetrical miR* expression or biased miRNA-5p/miRNA-3p expression.

Strand Reversal		Biased→Symmetrical		Symmetrical→Biased	
miR* >80% hairpin	Tags	miR*20–80% hairpin	Tags	miRNA	Tags
miR-140†	194,425	miR-322†	253829	miR-125a-5p†	511970
miR-674	45,917	miR-22†	48046	miR-345-5p	137955
miR-877	24,357	Let-7d†	27008	miR-542-3p	107193
miR-211†	19,925	miR-700	20139	miR-208a-5p†	91803
miR-330†	6,606	miR-361†	19126	miR-342-3p †	85185
miR-147	825	miR-872	17271	miR-151-5p†	66756
miR-879	792	miR-503	10254	miR-139-5p†	57793
miR-1943	752	miR-425	6643	miR-532-5p	57452
miR-463	747	miR-7a-1	6196	miR-339-5p	47424
miR-1935	612	miR-28†	5278	miR-423-3p†	36902
miR-3074-1	426	miR-3068	5128	miR-331-3p	34946
		miR-33	4954	miR-188-5p †	10962
		miR-676	3655	miR-450b-3p	10825
		miR-1981	2011	miR-671-5p	9195
		miR-132†	1679	miR-3096-5p	5304
		miR-96	1560	miR-1198-5p	2573
		miR-130b	1078	miR-878-3p	2259
		miR-465b-1	1007	miR-1843-3p	1654
		miR-463	1003	miR-511-3p	1429
		miR-190	747	miR-743b-3p	684

Thresholds were set at expression ≥150 tags (only miRNA mapped to one loci shown). Entries are ranked by tag abundance and truncated after the top 20 entries (except strand reversal).

†(pre-)miRNA with known function and/or expression in the heart as defined in [29].

Of the 77 examples with unexpected strand bias noted in HL-1 cells 27 are part of the ‘cardiac miRNA set’ [29] referred to above. To illustrate the potential biological relevance of strand bias to cardiac function we analysed gene function enrichment within Targetscan-predicted targets using Ingenuity pathway analysis, focusing on cardiac-related miRNAs and miRNAs with potentially cardiac-specific strand bias (Table S6). One important example already mentioned above is miR-22, which is involved in the cardiac hypertrophic response [34] and has an abundant miR* in HL-1 cells and the heart (58% and 40% of tags from hairpin respectively, Figure 2C). Interestingly, the predicted targets of miR-22* are significantly enriched for genes functioning in cardiovascular development, function and disease. This is similar to mature miR-22, although their predicted targets are different, suggesting both miRNAs regulate similar processes through different targets. Another likely cardiac-specific abundant miR* is derived from miR-322 (58%; Figure 2C), whose mature form is involved in myocyte differentiation [35], post-ischemic vascular remodeling and angiogenesis [36]. Analysis of predicted targets of miR-322 and miR-322* showed that they are significantly enriched for genes functioning in organ development and gene expression (Table S6), suggesting that both species are likely to play important roles in cardiac development and remodeling. The predicted targets of many other miRNA species with unexpected bias in HL-1 cells and the heart also have cardiac-related functions (Table S6), suggesting they have important roles in cardiac biology.

Thus, our results are consistent with the notion that one hairpin strand is typically chosen as the mature miRNA, and we broadly confirmed the mature/miR* and -5p/-3p annotations listed in miRBase up to version 17. Nevertheless, we also documented numerous examples of unexpected, and in part cardiomyocyte-specific strand bias.

### Numerous cardiomyocyte miRNAs exist as 5′ isomiRs, which can differ in their targeting properties

5.2**%** of all tags in our HL-1 dataset represented 5′ isomiRs, broadly similar to reports from other murine tissues and C. elegans [22], [37], and they were seen at a low level for virtually all miRNAs. Interestingly, 5′ isomiR tags were markedly more common on the 3′ compared to the 5′ hairpin arm (Figure 2A). For 105 miRNAs the proportion of tags mapping to 5′ isomiRs represented more than 20% of all tags (Figure 2D), with 22 represented by >80% of tags (Table 2). Of these 105 examples of high 5′ isomiR proportion, 58 were also detectable in the lower depth heart biopsy dataset and 42 of these again displayed >20% 5′ isomiRs tags. To assess the universality of 5′ start sites for these miRNA, we then reanalysed miRNA data from other murine tissues [22] and saw that, of the 105 examples, 12 had not previously been noted as they were not appreciably expressed in any of these other tissues (<10 tags; miR-1927, -1982, -3057, -504*, -1946b, -3079, -3076, -3082, -1949, -511, -2145-1, -1937c, the latter two were removed from miRbase version 17). For the remainder we required expression of the miRNA in at least 3 tissues and observed 55 miRNAs with high proportions of 5′ isomiRs (>20% of tags) in all tissues where they were expressed, suggesting that their 5′ isomiR proportion is consistently maintained in different cellular environments. 28 miRNAs had more variable levels of 5′ isomiR expression across tissues and/or embryonic stages (expression ≤20% in at least one other tissue; Table S7), while 5 examples consistently had ≤20% 5′ isomiR levels in all the other tissues. This suggests tissue-specific regulation of miRNA processing for the latter two groups and perhaps specific biological purpose for those 5′ isomiRs in cardiomyocytes.

**Table 2:** 5′ and 3′ isomiRs in HL-1 cardiomyocytes.

5′ isomiRs		3′ isomiRs	
>80% tags	Tags	>80% tags	Tags
miR-140*†	194425	miR-30d†	2281625
miR-133a-1*†	166829	miR-21†	728598
miR-101a†	36475	miR-30a†	499855
miR-504	34530	miR-30e†	487980
miR-183*	25076	miR-351†	207994
miR-145*†	7456	miR-181a-1†	196000
miR-1937a	4692	miR-140*†	194425
miR-1983	3964	miR-195†	158413
let-7g*†	2575	miR-3102	153943
miR-1957	1733	miR-345-5p	137955
miR-132*†	1679	miR-183	135000
miR-483†	695	miR-181b-1†	128529
miR-3082-5p	479	miR-542-3p	107193
miR-222*†	469	miR92a-1	105635
miR-670*	436	miR-497†	76818
miR-1186	342	miR-16-1†	70920
miR-1949	278	miR-206	60867
miR-3470b	261	miR-339-5p	47424
miR-511-5p	164	miR-15a†	40961
miR-3087	161	miR-350	38521

Thresholds were set at expression ≥150 tags (only miRNA mapped to one loci shown). Entries are ranked by tag abundance and truncated after the top 20 entries.

†(pre-)miRNA with known function and/or expression in the heart as defined in [29].

Importantly, within the cardiac miRNA set [29], 35 had a 5′ isomiR proportion of >20% (8 of these >80% of tags). To investigate the biological role of these miRNAs and their 5′ isomiRs we used their respective seed sequences for target predictions and found highly individual relationships between mRNA targets and enriched gene function classification (Table S8). For example, 83% and 96% of miR-133a* tags were contributed by a -1 5′ isomiR in the HL-1 and heart biopsy datasets, repectively. Importantly, unlike the canonical variant, the 5′ isomiR is predicted to target numerous mRNAs involved in cardiovascular disease (Figure 2F). Conversely, individual and common predicted targets of miR-101a and its -1 5′ isomiR (87% and 74% of HL-1 and heart biopsy tags, respectively; also predominant in ES cells but not the liver [38]) are strongly implicated in cardiovascular disease, suggesting they may act through both common and distinct targets to affect cardiac function (Figure 2F).The predicted targets of miR-100* and a prevalent 5′ isomiR derived from a +1 position (57% and 77% of HL-1 and heart biopsy tags, respectively) have similar proportions of targets functioning in cardiovascular development. Overlapping targeting and functions are also observed for predicted targets of miR-222* and its -1 5′ isomiR (81% and 31% of HL-1 and heart biopsy tags, respectively), while little overlap was observed in the targets of miR-140* and its +1 5′ isomiR (86% and 79% HL-1 and heart biopsy tags, respectively), though the enriched functions of their targets is remarkably similar (Gene function analysis in Table S8).

In summary, we found variation of the 5′ start site of miRNAs to be relatively rare in cardiomyocytes. Nevertheless, many individual miRNAs showed high proportions of 5′ isomiR expression in cardiomyocytes. The proportion of 5′ isomiR was relatively constant for some miRNAs but appeared to be under tissue-selective/development-specific control for others, and is likely to affect their biological function.

### 3′ isomiRs are common in cardiomyocytes

52.3% of the generic miRNA tags in the HL-1 dataset indicated miRNA variability at the 3′ end, which is qualitatively similar to other systems [21], [22], [37]. 379 miRNAs were represented by >20%, and 112 miRNAs by >80% of tags with alternate 3′ ends from those described in miRbase (Figure 2E; Table 2). Similarly, 316 of 381 detectable miRNAs in the heart biopsy had 3′ isomiRs at >20% tag proportion and 3′ end heterogeneity was likewise common in other tissues [22]. The modal tag length for all generic miRNAs in HL-1 cardiomyocytes was 22 nt, although tags deriving from the 5′ arm were 22 or 23 nt long in approximately equal proportions (Figure 1B).

Unambiguous 3′ additions, as well as internal sequence changes, were relatively rare in the HL-1 dataset (Text S1, Figure S4 and Table S9). In datasets from other tissues non-templated additions were biased to A and U extensions [22], [39], but we did not observe any nucleotide bias or commonly extended miRNAs in cardiomyocytes (>5% tags). Instead the most detectable 3′ end variation in our data appears to be the result of altered 3′ cleavage or trimming events. There were three major types of 3′ end variability observed in our dataset (Figure 2G); a small range of 3′ end sites (most common, e.g. miR-499); a large spread of 3′ end sites used, perhaps indicating 3′ trimming (e.g. miR-181a); or a defined 3′ end site, albeit not necessarily as annotated in miRBase (e.g. miR-15a). In all cases there are clear preferences for some sites, suggesting regulated alternate cleavage or trimming events. Importantly, most members of the cardiac miRNA set had >20% of mapped tags with 3′ ends that differed from miRBase (177 of 181 that were appreciably expressed; 40 miRNAs with >80% of 3′ end variant tags). Three notable examples include: miR-499, a MyomiR that controls myosin heavy chain isoform expression [4] and is a biomarker of myocardial infarction [40]; miR-21, which is currently touted as a promising therapeutic target for cardiovascular diseases [41]; and miR-195, whose deletion in mice leads to cardiac hypertrophy [5]. We find abundant, if not predominant, species of all these miRNA that are one base longer than annotated in miRBase in both the HL-1 cardiomyocyte and heart biopsy datasets.

A related observation was that although 90% of all tags were 24 nt or shorter (Figure 1B), 28 miRNAs had >20% of tags that were longer than 24 nt (Table S9), the same was found with 8 of 11 detectable cases in the lower coverage heart biopsy dataset (see Text S1 for structural analysis of long miRNAs). miR-301a is an extreme and abundant case, with >60% of tags being 24 nt or longer in cardiomyocytes (Figure 2G, Figure S5A), which we confirmed by northern blotting (Figure S5B). Re-analysis of other murine tissues [22], [42] additionally suggested that the length of this miRNA is tissue-specific and developmentally regulated (Figure S5C). Interestingly, both miR-301a and several members of the miR-30 family, which are also commonly longer than 24 nt in our dataset (Table S10), target the mRNA for plasminogen activator inhibitor-1, a protein involved in the pathogenesis of cardiovascular disorders [43]. Another prominent example is miR-181a, which is involved in cardiovascular development [44], yet 47% of tags are longer than 24 nt in our cardiomyocyte dataset.

In summary, we find that miRNA 3′ end variation is common in cardiomyocytes and many miRNAs with established cardiac functions are altered in this way.

### Expression of 5′ isomiRs can alter the target spectrum of miR-133a

**Figure 3:**
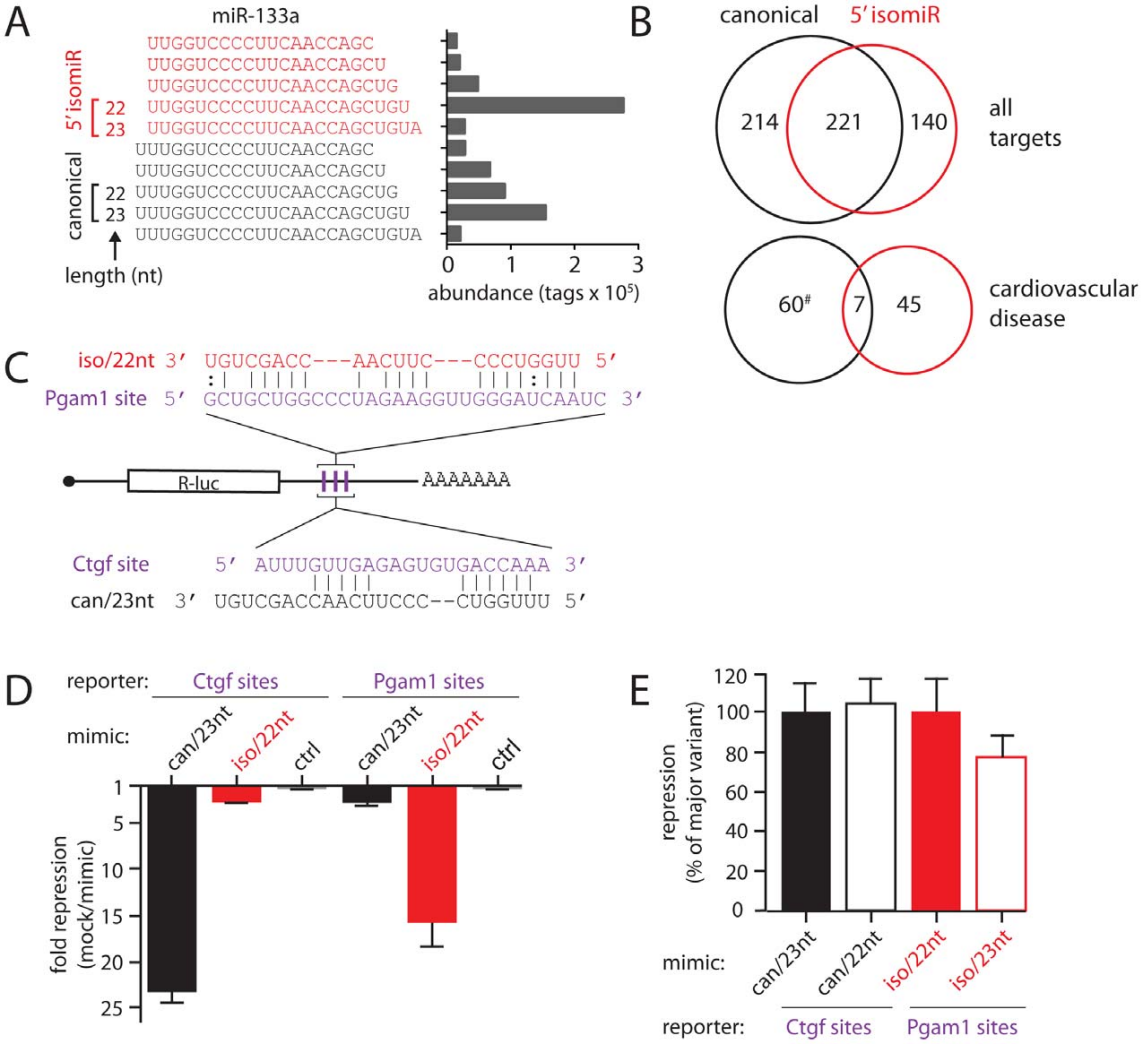
isomiRs of miR-133a with different targeting properties. (**A**) Major mature miR-133a species and their abundance in HL-1 cardiomyocytes. Sequence tags are grouped into those with canonical (black, ‘can’) and +1 (red, ‘iso’) 5′ start sites. Brackets denote sequences used as miRNA mimics in panels D and E. (**B**) Venn diagrams indicate number and overlap of predicted mRNA targets (Targetscan) for canonical and +1 5′ isomiR variants of miR-133a (top: all predicted targets, bottom: only targets with roles in cardiovascular disease (Ingenuity; #: significant enrichment of gene function term, p<0.01). (**C**) Schematic of reporter constructs made to contain three copies of predicted miR-133a binding sites from *Pgam1* or *Ctgf* mRNA 3′UTRs. Base pairing potential between sites and miR-133a isomiRs is also shown. (**D**) *Ctgf* or *Pgam1* R-luc reporters were transfected into HeLa cells with mimics of the major variants of the canonical (can/23 nt) and +1 5′ variants (iso/22 nt) of miR-133a, an irrelevant control, or no mimic at all, and luciferase activity measured 24 hours later. The fold change of expression is calculated as no mimic/mimic and results are averages of four independent experiments with standard error. (**E**) Transfections as in (D) comparing mimics of different lengths (can/23 nt vs can/22 nt, iso/22 nt vs. iso/23 nt; for sequence see panel A). Repression is given as a percentage of that seen with the respective major variant and results are averages of at least three independent experiments with standard error.

miR-133a is known to have key roles in cardiac biology [45], [46], [47] and its example further illustrates the diversity of hairpin precursor processing. We saw expression of 5′ isomiRs from both strands of the hairpin (a prevalent 5′ isomiR of miR-133a* is shown in Figure 2F), and we next focussed on mature miR-133a. In addition to tags with the canonical 5′ start position, we detected a highly abundant +1 5′ isomiR of 50.1**%** and 53.9% of mature miR-133a tags in HL-1 cells and the heart, respectively (Figure 3A), similar to that seen in the heart on another sequencing platform [22]. In mammals it is known that miR-133a 5′ isomiRs can be derived from two identical genomic loci, named miR-133a-1 and miR-133a-2, and thus these isomiR proportions are not likely due to processing differences of individual loci [22]. Furthermore, we also observed that both 5′ start positions were associated with variable 3′ ends (Figure 3A). We used the respective seed sequences (base 2–8) of the miR-133a 5′ isomiRs to predict mRNA targets using TargetScan (Figure 3B). Overall, this analysis yielded both common and unique targets, with both 5′ isomiRs being predicted to target many genes involved in cardiovascular disease, although few of the latter were common to both. This suggested that both 5′ isomiRs might regulate similar cardiomyocyte functions largely through different mRNA targets.

To experimentally test if the 5′ isomiRs of miR-133a can have different targeting properties *in vivo*, we created two luciferase reporter constructs, each carrying three copies of a different predicted miR-133a target site in their 3′ UTR (Figure 3C). These sites were derived from two experimentally demonstrated targets of the miR-133a locus, the *Ctgf* and *Pgam1* mRNAs [48]. The reporters were transiently transfected into HeLa cells together with synthetic RNA mimics of the two most abundant mature miR-133a variants, a canonical 5′ start site variant of 23 nt (can/23 nt) and a +1 5′ isomiR of 22 nt (iso/22 nt; Figure 3A), or a mimic of an unrelated miRNA. This revealed pronounced preferential targeting by each variant such that there was significantly greater repression of the *Ctgf* reporter by canonical miR-133a than the isomiR, and *vice versa* for the Pgam construct (Figure 3D). Given that the two mimics chosen varied in length (22 *versus* 23 nt), we tested two further mimics representing the respective other length for each miR-133a variant (can/22 nt and iso/23 nt) and found that repression did not depend on mimic length within this range or vary significantly between different 3′ end sites (Figure 3E).

Thus, mRNA target predictions for major 5′ start site variants of miR-133a suggested convergent biological function at least in part through divergent target spectra. Addressing this issue experimentally, we have also been able to provide proof-of-principle evidence for alternative mRNA targeting by the major canonical and major 5′ isomiR variants of miR-133a in living cells.

### Sequence and structural features of miRNA precursors

miRNA processing is largely determined by DGR8/Drosha and Dicer recognizing structural elements in their RNA substrate (ssRNA/dsRNA junction and Drosha cleavage site, respectively) and then cleaving at a set distance away from it [13], [49]. Nevertheless, local sequence context and structural distortions are also known to contribute to cleavage site selection [14], [37], 5′ end nucleoside identity and base pairing affect RISC loading and trimming of miRNA 3′ ends further affect which miRNA sequences will accumulate in cells [50], [51], [52], [53].

**Figure 4:**
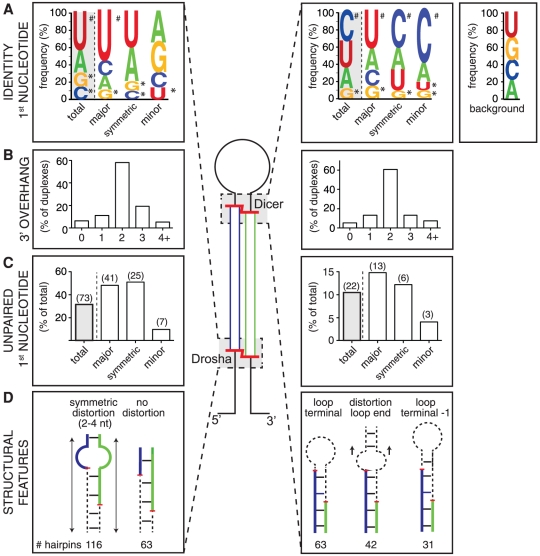
Sequence and structural features around cardiomyocyte miRNA termini. (**A**) Sequence logos displaying nucleoside frequency at the 5′ start position of miRNAs residing on the 5′ or 3′ hairpin arm. Results are shown averaged over all miRNAs (total), or after grouping miRNAs into asymmetrically expressed major and minor species (≥80% or ≤20% of tags from the hairpin, respectively) as well as symmetrically expressed miRNAs (less than 80%, but more than 20% of tags on any one arm of the hairpin). Significant over- (#) and under-representation (*) compared to background is also shown (p<0.01, t-test with Welch adjustment). Background was defined as the average of positions adjacent to all cleavage sites. Only generic miRNAs with expression level ≥150 tags were included in this analysis. See Figure S6 for expanded analysis. (**B**) Proportion of miRNAs with different 3′ overhang lengths in reconstructed duplex processing intermediates. (**C**) Proportion of miRNAs with an unpaired first nucleoside after grouping miRNAs as in (A). Number of miRNAs in each group is shown in brackets above. (**D**) Shown are schematics of the most common structural distortions on miRNA precursors surrounding miRNA termini (see Figure S7 for details). Analyses in (B–D) were performed on canonically processed miRNA hairpins with expression level ≥150 tags from at least one arm. Structural features were determined by visual inspection of the most common isomiR on each arm overlaid onto the predicted miRNA hairpin folding as deposited in miRBase.

We therefore examined the first and last positions in HL-1 cardiomyocyte miRNA sequence tags as well as at the immediately adjacent external positions and calculated nucleoside frequencies for 403 appreciably expressed generic miRNAs (see Materials and Methods for further details). This revealed a significant enrichment (p<0.01) of uridine at the first and last position within miRNAs derived from the 5′ arm of hairpins, as well as at the 5′ most position of the excised terminal loop segment, and the first position of miRNAs derived from the 3′ arm was enriched for cytidine (Figures 4A; see Figure S6 for a full display of results). Other nucleosides, primarily cytidine and guanosine, where significantly depleted at these positions, relative to background nucleoside composition. We also recorded nucleoside frequencies after grouping miRNAs into asymmetrically expressed major and minor species (≥80% or ≤20% of tags from the hairpin, respectively) as well as symmetrically expressed miRNAs (less than 80%, but greater than 20% of tags on any one arm of the hairpin). Focussing on the 5′ start of miRNAs, we observed a strong preference for uridine at this position for major miRNA species derived from both arms of the hairpin (Figure 4A). Conversely, uridine was strongly depleted from the 5′ most position of the minor miRNA species, again on both hairpin arms. We found a strong preference for cytidine at the 5′ start of minor miRNA species deriving from the 3′ arm of hairpins. Symmetrically expressed miRNAs on both arms exhibited 5′ nucleoside bias that was intermediate between those of the corresponding major and minor species. Overall, our analyses indicate that there is no absolute requirement for a given nucleoside at any of the positions we examined. Nevertheless, the observed patterns of enrichment and depletion of nucleosides are consistent with a role for local sequence context in miRNA processing and/or incorporation into argonaute proteins. In particular, the pattern of uridine bias at the miRNA 5′ end is consistent with the known bias towards this nucleoside at the first position of miRNAs and the preference of mammalian Ago 2 for uridine or adenine in this position [50], [51]. While the frequency of adenine varied between miRNA categories, any bias for or against it was comparatively minor. A curious finding was the strong preference for cytidine in the 5′ most positions of minor and symmetrically expressed miRNA species only on the 3′ arm, suggesting factors beyond discrimination by Ago 2 are at play in this case.

With the proviso that miRNA start and end positions as measured by sequencing are subject to trimming as well as ‘purifying’ Argonaute selection, we further examined local RNA structure near processing sites in 229 appreciably expressed and canonically processed hairpins (196 with expression from both arms), considering only the most abundant expressed isomiR variant on each arm. We noted that a majority of miRNA duplexes reconstructed in this way had a 2 nucleotide overhang at Drosha- and Dicer-processed ends (58.1% and 60.7%, respectively; see Figure 4B), consistent with the known property of both enzymes to produce recessed termini in this way, but also significant post-processing extension and trimming of miRNA 3′ ends. Next, we determined the extent of base-pairing of the 5′ most position of miRNAs and saw two clear tendencies. We found that a majority of miRNA duplexes were base-paired at both ends (68.4%). Beyond that, there was a preference for the first base of miRNAs residing on the 5′ arm of the hairpin to be unpaired, compared to those residing on the 3′ arm (31.5% and 10.5%, respectively; see Figure 4C). When considering major/minor and symmetrically expressed species separately, additional trends emerged. First, regardless of arm location, there was a bias towards the major miRNA species exhibiting an unpaired 5′ terminus, although this effect was much more pronounced when major species resided on the 5′ arm than on the 3′ arm (48.2% and 14.8%, respectively). Second, minor species tended to favor base-pairing of their 5′ termini; this effect was particularly striking with minor species residing on the 5′ arm, where only 9.5% of minor species had an unpaired terminus, compared to 31.5% of all miRNAs on that arm. Third, with symmetrically expressed hairpins, miRNAs on both arms exhibited virtually the same preference for unpaired 5′ ends as seen with major species in these locations, i.e. there was a bias towards unpaired 5′ termini at both ends of the miRNA duplex. Overall, these results indicate that there is no absolute requirement for an unpaired 5′ terminus to allow a miRNA to accumulate. Nevertheless, we detect a clear signature of the established thermodynamic stability rule for miRNA strand selection [33]. The general bias for an unpaired miRNA 5′ terminus on the 5′ arm of hairpins can be explained by more common structural distortions around the Drosha cleavage site compared to the Dicer cleavage region (see below).

Next, we extended our analyses to assess more generally local structural distortion around miRNA processing sites. We found that 63 Drosha cleavage regions were base-paired throughout. The majority of Drosha cleavage sites featured some form of structural distortion. Most common was a symmetrical internal loop of 2 or 4 nucleotides (72 and 36 examples, respectively; see Figure 4D). The remainder of sites (58) had miscellaneous other structural distortions (see Figure S7 for a full analysis). The most common structure near the Dicer cleavage site was the terminal loop region, with the 5′ arm cleaved immediately prior to the loop for 63 hairpins and 1 nt into the stem for a further 31 (Figure 4D). The majority of these miRNAs had no other structural distortions in the vicinity of the Dicer processing region (54 and 28 hairpins, respectively). The next most common structural feature near the Dicer cleavage site were smaller bulges and loops starting immediately after the 5′ arm cleavage site (42 hairpins, Figure 4D). There was no preference for asymmetrical structures to be on the 5′ or 3′ arm for either cleavage site (data not shown). Altoghether, these findings reveal that there is no absolute requirement for specific structural features around either cleavage region. Nevertheless, there was a tendency towards symmetrical structural distortions around and within in the Drosha cleavage region. For Dicer, the immediate cleavage region typically was base-paired but immediately adjacent to either the terminal region loop or other distortions that may be functionally equivalent.

### Novel and unusual miRNAs originating from known miRNA precursors

**Figure 5:**
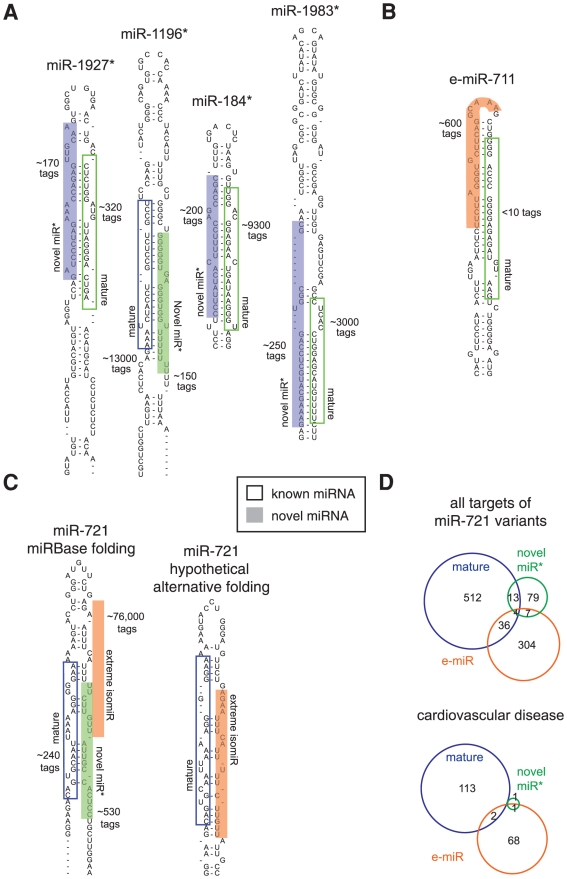
New miRNA variants mapping to known hairpin precursors. (**A**) Examples of generic miR* species discovered in HL-1 cells not currently annotated in miRBase. Position within the hairpin structure is consistent with canonical processing. (The additional case of miR-721* is shown in panel C). (**B**) We refer to a set of miRNA tags outside the canonical processing region of hairpins as an extreme isomiR (e-miRs). Position of the major e-miR on the mir-711 hairpin suggests it as a novel case of Ago2-mediated processing [54]. (**C**), The miR 721 hairpin gives rise to an abundant e-miR; expression of the annotated mature miR-721 and its generic miR* counterpart is much lower. *Left,* hairpin fold as listed in miRbase; *right*, hypothetical alternative fold consistent with canonical processing of miR-721 together with the novel e-miR-721*. (**D**) Venn diagrams indicate number and overlap of predicted mRNA targets (Targetscan) for the three miRNA variants mapping to the miR-721 hairpin (top: all predicted targets, bottom: only targets with roles in cardiovascular disease (Ingenuity). miRNAs already annotated in miRBase are marked by open boxes, while novel species are highlighted by filled boxes.

To search for evidence of novel miR* we looked for tags that mapped to hairpins opposite to known mature miRNA. After applying a ≥150 tag expression threshold we retained 5 examples of novel generic miR* (miR-1927*, -1196*, -184*, -1983* and -721*; Figure 5A & C, Table 3), whose position is consistent with canonical Drosha/Dicer processing of the respective miRBase-annotated miRNA hairpin structure. In the recent release of miRBase v18 miR-184 and miR-1196 hairpins have miRNA annotations on both arms, although these new miR*s were not derived from experimental evidence. Furthermore the sequence of miR-1196 is not the same as our observation, nor compatible with canonical processing of the predicted structure, suggesting that our miR-1196* is a more appropriate miR* candidate.

**Table 3:** Novel miR*, novel non-canonical miRNAs and novel antisense miRNA.

Novel miR*		Non-canonical miRNAs	
miRNA	Hairpin%†	miRNA	Hairpin%
miR-1944*||	68.8	e-miR-721*	99.7
miR-3470b*	47.8	e-miR-3072	99.97
miR-1983*	generic	e-miR-1274a||	96.06
miR-1187*	99.1	e-miR-1983	54.22
miR-3471-1*	99.6	e-miR-1937b-1||	46.77
miR-721*	generic	e-miR-1947	97.55
miR-467f*	96.6	e-miR-1933-5p	77.40
miR-1195*	72.2	e-miR-1934*	73.05
miR-2145-1*||	82.8	e-miR-136	98.31
miR-678*	99.8	e-miR-711	98.8
miR-1927*	generic	e-miR-690	92.78
miR-688*	92.1	e-miR-3473	45.87
miR-1196*	generic	e-miR-463	48.4
miR-184*‡ ∧	generic	e-miR-697	97.08
miR-717*	98.2	e-miR-194-1*‡	54.74
miR-1892*	92.5	e-miR-592	92.13
		e-miR-344g-5p	97.08
Antisense miRNAs§		e-miR-455*	68.65
miRNA	Counts	e-miR-2137	30.87
miR-30e-as‡	5,996	e-miR-144	89.86
miR-873-as	1,016	e-miR-743b-5p	23.75
miR-449c-as	515	e-miR-715||	69.35
miR-541-as	439	e-miR-881*	56.21
miR-148b-as	336	e-miR-370	97.41
miR-546-as	333	e-miR-3067	100
miR-3074-as	262	e-miR-448-5p	100
miR-451-as‡	286	e-miR-669o-5p	99.35

† Novel miR* that are processed within the expected window of the mature strand are labelled “generic”. Entries are ranked by tag abundance.

‡ (pre-)miRNA with known function and/or expression in the heart as defined in [29].

§ All antisense hairpins have at least one tag aligned to the opposing side of the stem.

|| miRBase v16 annotated miRNAs removed from miRBase v17.

∧ reported in miRbase v18.

Because there is precedent for a number of unusual processing events that can give rise to miRNAs, we then extended our analysis to the 266,989 miRBase-mapped tags whose 5′ ends fell outside the expected start position of a generically processed miRNA (more than ±3 nt) and further added 3,594 tags that mapped to the mouse genome just outside the miRNA hairpin sequences listed in miRBase. This allowed detection of two highly expressed contiguous sets of miRNA pairs from the long stem of the putative miRtron mir-3102 (Dataset S1 and S2) [22]. Another known example of unusual pri-miRNA processing we detected was miR-451, which overlaps with the hairpin loop and is generated by a Dicer-independent, AGO2-dependent mechanism [54]. Interestingly, we found a novel variant on the miR-711 hairpin (Figure 5B), which we termed an extreme isomiR (e-miR-711; see Figure S3 for an explanation of miRNA variant nomenclature used here). e-miR-711 is the major species mapped to this hairpin in the HL-1 and heart biopsy datasets and may similarly be derived by AGO2-dependent processing.

To search for any other high confidence e-miR variants we further required that non-generic sequence tags represented ≥20% of the total tags mapped to a given miRNA hairpin. These criteria retained 63 hairpins for further visual inspection (Table S11). 25 of these were disqualified on the basis of a lack of credible miRNA-like properties. As a plausible explanation for the genesis of the remaining 38 examples we considered that many may arise from generic Drosha/Dicer processing of pri-miRNAs adopting a hairpin fold *in vivo* that differs from the miRBase-prediction. With that proviso we assign 11 tag sets as representing novel non-canonical miR*. (Interestingly, several examples of this already exist in miRbase, e.g. miR-147, miR-3096 and miR-3113). 27 tags sets mapped to a hairpin arm that already harbours a miRBase-annotated miRNA species, thus we classify them as additional novel e-miRs (Table 3). Many of the unusual miRNA processing events described above were also seen in the lower depth heart biopsy dataset. Specifically, of the 17 detectable miRNA hairpins, 14 had observable e-miRs and 10 of these were expressed in similar proportions as in HL-1 cardiomyocytes. Four of the pre-miRNAs with observed novel miR* had some coverage in the lower depth heart biopsy dataset, and thus we could confirm expression of the novel miR-1944*, miR-3470* and miR-1195* (Dataset S2).

e-miR-721* is the most abundant non-generic miRNA in the cardiomyocyte and heart biopsy datasets (Figure 5C). As an indication of the biological significance of e-miR-721*, we performed gene function analysis with the predicted targets of the three miR-721 hairpin-derived miRNAs. The mature and e-miR-721* are predicted to target appreciable numbers of genes involved in cardiovascular disease, unlike the novel generic miR*, suggesting a reason for the processing of the e-miR-721* to be favored in cardiomyocytes (Figure 5D). Furthermore, there is much greater sequence identity across species for e-miR-721* than mature miR-721, suggesting this to be the more important miR-721 variant expressed at this locus (data not shown).

In summary, our data suggests that unusual miRNA variants are frequently derived from their precursors, many with potentially important cardiac functions. Regulated modulation of hairpin folding may underlie many of these observations.

### Antisense miRNAs and other novel miRNAs

**Figure 6:**
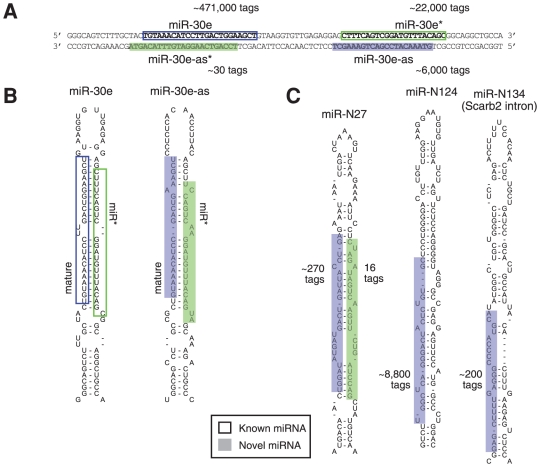
miRNAs candidates deriving from novel precursors and genomic locations in HL-1 cells. (**A**) The *miR-30e* locus appears to be expressed bi-directionally, giving rise to miRNAs tags sets mapping to both, the sense (known) and antisense strands (novel; suffix –as denotes antisense-derived miRNA). (**B**) Predicted structures for both sense and antisense miR-30e hairpin precursors. (**C**) Examples of tag sets mapping to entirely new candidate miRNA loci in the murine genome. These miRNA species are tentatively named miR-N.. (N for novel). Predicted hairpin structures (RNALfold) of surrounding sequence is shown.

Next, we analysed our dataset for the presence of miRNAs that are derived from precursors transcribed in antisense orientation to known miRNA loci [55] using ∼600,000 tags that mapped to the opposite strand of miRBase-listed hairpins. We also looked for expression of entirely novel miRNAs in 23,953,195 tags that mapped to the mouse autosomes and X chromosome. Using a bioinformatic pipline to identify abundant tags in genomic regions with properties of known miRNA (described in Text S1 and shown in Figure S8) we identified 8 putative antisense (as) miRNAs (Table 3) and 147 genomic regions corresponding to putative novel miRNA precursors (Dataset S3), with individual tag counts of up to 8748. Thirteen of the novel candidate miRNAs (termed ‘miR-N’ plus a serial number), and all of the antisense miRNAs, had expression of a miR* form (e.g. miR-N27 and miR-30e-as; Figure 6), providing additional confidence in suggesting these as a *bona fide* miRNA. Furthermore, we have verified the expression in HL-1 cells of two novel miRNA and their repective miR* (miR-N4/miR-N4* and miR-N29/miR-N29*) and one antisense miRNA (miR-30e-as) by high stringency PCR with melt curve analysis (Text S1 and Figure S9).

39 novel miRNAs had a seed sequence identical to a known miRNA family (Dataset S3). For example, six novel miRNAs share their seed sequence with the cardiac-related miR-1/206 family. While seven novel miRNAs originated from snoRNA-encoding loci, 64 others were derived from protein coding genomic loci. 33 of the latter mapped to the sense strand, with 31 being situated in introns. Furthermore, 17 novel miRNAs were derived from gene loci associated with in cardiac biology (as defined by Ingenuity, e.g. *Gnaq*, *Ntn1*, *Rttn*, *Gli2*, *Man1c1* and *Scarb2*).

Our novel miR-N108, which is encoded in an intron of *Eif4g3*, has been annotated as a *bona fide* miRNA in miRBase version 17 (now called miR-5123 [56]), but none of the other 134 entries new to version 17 and 18 are related to our novel miRNAs. None of our novel miRNAs have been previously removed from miRBase or, to our knowledge, been detected in other recent deep sequencing publications. There are also no already annotated orthologs in conserved syntenic genomic regions in other species (data not shown). However, novel mir-N84 has been bioinformatically predicted and is listed in Deepbase [57]. Additionally, while a number of antisense miRNA are known (including miR-1-2as now called miR-1b, which we did not detect here), none of the antisense miRNAs reported here, to our knowledge, have been previously observed. Thus, while we have support for our novel miRNA discovery pipeline through some overlap of its output with predicted and experimentally verified miRNAs, the bulk of our novel candidate miRNAs are without precedent.

Of the 8 identified novel antisense miRNAs present in our dataset, miR-30e-as is the most abundant and arguably the most interesting (Figure 6A&B, Figure S9). The sequences for mature miR-30e and miR-30e-as are notably similar, and miR-30e-as is relatively abundant in the heart biopsy dataset at ∼4% of miR-30e expression. The predicted targets of miR-30e and miR-30e-as overlap considerably and there is significant enrichment of targets involved in cardiovascular disease for individual and overlapping predicted targets (67/283 common, p<0.01; 154/648 miR-30e only, p<10^−6^; and 88/456 miR-30e-as only, p<0.5). Furthermore miR-30e is known to be commonly down-regulated in hypertensive heart disease [46] and regulates connective tissue growth factor in myocardial matrix remodelling [47], suggesting miR-30e and (by association) miR-30e-as have important roles in cardiac biology.

In summary, by applying stringent criteria to a dataset of great depth we have found expression of many candidates for antisense and entirely novel miRNAs in cardiomyocytes.

## Discussion

We presented here a comprehensive census of the murine cardiomyocyte miRNA repertoire. Detailed information down to sequence tag level for each miRNA is available in Table S1 and Dataset S1. We saw appreciable expression of 403 known miRNAs, as well as detecting five novel miR*, 38 extreme isomiRs, eight antisense miRNAs, and 147 entirely novel miRNA candidates. We furthermore documented in detail the contribution of processing variants to the miRNA population, describing 77 hairpins exhibiting unexpected strand bias and 105 examples of high 5′ isomiR proportion, while most miRNAs exhibited variable 3′ ends. Importantly, 55 miRNAs derived from hairpins with known cardiac function [29] exhibit one or several of these unusual features. Often for these miRNA (e.g. 9 cases of unexpected strand bias and 15 instances of high 5′ isomiR proportion) the unusual feature was not as prominant in other murine tissues previously analysed [22], suggesting particular relevance to cardiomyocyte biology. We then focused on abundant 5′ isomiRs and directly demonstrated potential for differential mRNA targeting for two 5′ isomiRs of the key cardiac regulator miR-133a. More broadly, we found that 55 of 105 5′ isomiRs were prevalent (>20% of tags) also in all other murine tissues where they were appreciably expressed [22]. Of the remainder, 12 were below detection limits in non-cardiac tissue and 28 exhibited varying degrees of tissue-selective accumulation, suggesting frequent tissue-specific miRNA processing and cardiomyocyte-specific roles for a subset of 5′ isomiRs.

### Features of miRNA biogenesis and function

Our dataset confirmed the established view that most miRNA hairpins are asymmetrically processed, yielding mature miRNAs predominantly from one arm. Concordance with miRBase version 16 assignment of mature and miR* was generally good and we also found that -5p/-3p annotated miRNAs tended to be expressed relatively evenly (Figure 2B). Nevertheless, expression was detectable at some level from both arms of most hairpins and, like others [33], [58], we found that many individual hairpins markedly deviate in strand bias from their miRBase version 16 annotation. In some cases this appeared to reflect cardiomyocyte-selective miRNA processing, in others we observed similar arm bias upon reanalysis of datasets from other tissues [22]. Notably, the recently released miRBase version 18 has dispensed with the mature/miR* nomenclature and instead renamed all murine miRNA species with a -5p or -3p suffix, acknowledging the notion that (regulated) expression from both arms can give rise to functional miRNA species [59], [60]. It is now appreciated that multiple miRNA features may affect miRNA strand selection (reviewed in refs [11], [12], [14]). We found that no single aspect was uniquely required, however, two known features were sufficiently common to leave a ‘signature’ in our analyses. First, using an unpaired 5′ base as a surrogate measurement (Figure 4C), we saw patterns consistent with the established thermodynamic stability rule for asymmetric miRNA incorporation into miRISC [61], [62], [63]. Second, we could detect a clear overrepresentation of uridine at the 5′ start of major miRNA species (Figure 4A), consistent with the known bias towards this nucleoside at the first position of miRNAs and the preference of mammalian Ago 2 for uridine or adenine in this position [50], [51].

While the modal miRNA tag length in cardiomyocytes was 22 nt (Figure 1B), we observed common miRNA end heterogeneity, which was much more pronounced at the 3′ end than at the 5′ start site (52.3% and 5.2% of tags deviated from miRBase annotation, respectively; Figure 2D&E). As observed previously, heterogeneity at both termini was also less pronounced at the Drosha compared to the Dicer cleavage site (Figure 2A), perhaps suggesting more a precise cleavage by Drosha, or indicating a cumulative effect of variability in successive processing steps [64]. Additionally, it is clear that observable miRNA 3′ ends in our dataset did not strictly follow 5′ start choice, and thus much of the variability at the 3′ end probably derives from post-processing addition and trimming events [52], [53]. This is likely further reflected in our analysis of miRNA duplexes as we find that a large minority do not conform to the 2 nt 3′ overhang rule (Figure 4B). Similar trends were seen in previous analyses [22], [38], [65], and the basis for some of the diversity of miRNA termini and length has been ascribed to structural features of the pre-miRNA [14]. Additionally, proteins associating with Drosha or the hairpin loop sequences are known to regulate individual hairpin processing [11], [13], [37], [66], implying that many instances of end variability will be cell context-specific. Our data indicated a tendency towards symmetric structural distortions around and within in the Drosha cleavage region, while for Dicer, the immediate cleavage region was typically base-paired but immediately adjacent to either the terminal loop or other local distortions (Figure 4D). Similar observations were made for *C. elegans* Drosha and Dicer sites [37]. These structural features, while not strictly required, may contribute to miRNA processing accuracy, in addition to the in-built ‘ruler’ functions of the Drosha and Dicer complexes [49].

Importantly, in our dataset we observe high levels of variability for numerous individual miRNAs (Figure 2D&E), which are likely to impact on their role in cardiac biology. Evidence in favor of this notion includes the observation that isomiR expression is regulated during development [21] and variations at the 3′ end can influence miRNA stability [67], loading into distinct Argonaute complexes [16], [17], as well as potentially affecting target mRNA binding [20] and the cellular location of miRNAs [68]. There is also the strong expectation that 5′ isomiRs will have distinct mRNA targeting properties, since changes to the miRNA 5′ end will alter their seed region identity [21], [22], [38], [65]. Importantly, we present here a direct experimental validation of differential mRNA targeting by 5′ isomiRs using the example of miR-133a (Figure 3). Interestingly, alteration to the 3′ end of miR-133a mimics did not affect the level of mRNA repression, suggesting that in this instance the 3′ end is not essential for efficient target binding. Our work further provides information on expression of novel miR* (e.g. Figure 5A), a new candidate for AGO2-mediated processing (Figure 5B) and antisense-miRNAs (e.g. Figure 6A & B), as well as documenting a substantial repertoire of entirely novel miRNA candidates (e.g. Figure 6C). Remarkably, we also described numerous examples of extreme miRNA variants (e-miRs; e.g. Figure 5C), whose existence may often be explained by the pri-miRNA adopting an alternate secondary structure(s) that differ from the predicted structures deposited in miRBase. We therefore suggest that many novel e-miRs may be produced by canonical Drosha and Dicer processing of alternate miRNA harpin structures. While novel as a concept in this context, it is in general well established that long-range interactions within RNA molecules or interactions with cellular proteins can affect local RNA secondary structure (e.g. RNA chaperones [69]) and the current catalog of known miRNA hairpin interacting proteins is rapidly expanding [13].

### Implications for cardiac biology and disease

The HL-1 cell line is a popular cell culture model of cardiomyocyte biology and, as shown previously for transcriptomic and phenotypic aspects [30], we have demonstrated here that they express an adult cardiomyocyte-like miRNA profile. Furthermore, a small number of miRNAs with prominent roles in cardiac biology represented the bulk of the HL-1 cell tag count, as is typically seen in differentiated cells [70]. Nevertheless, the miRNA expression profile of HL-1 cells did deviate from a previously described whole heart miRNA expression profile [31] and our own left ventricle dataset in some respects, e.g. exhibiting higher miR-145 and lower miR-1 expression. This may partly be due to the transformed nature and cell culture environment of HL-1 cells, or their atrial origin as there is some evidence that expression of miRNAs differs between atrium and ventricle [32]. Importantly, our observations of the many miRNA processing variants were remarkably consistent between HL-1 cells and our ventrical biopsy. It was interesting to test whether the characteristic change from a non-beating to a beating state mimicked a distinct step in cardiomyocyte differentiation, however, the relative lack of differential miRNA expression we found (Figure S2) argues against this notion. Instead, we saw a bulk up-regulation in miRNA level, consistent with observations in other cells types reaching confluency [26]. It remains to be tested whether this merely correlates with the beating state, or directly contributes to it in some way.

The importance of miRNAs for cardiac development and cardiomyocyte function is well described [2], [3]. Many individual miRNAs have further been implicated in the pathology of heart disease, already leading to efforts of utilizing this knowledge for therapy development [2]. However, many of these studies were devised, conducted and their findings interpreted without detailed knowledge of the prevalence of miRNA sequence variation. The present study now provides such detailed information, which will at a minimum allow a more sophisticated understanding of previously generated data. For instance, knowledge of all extant 5′ isomiRs of a given disease-associated miRNA will enrich our picture of how they target the cardiomyocyte mRNA population and are thus involved in the disease pathology. miR-133a, a miRNA crucial to cardiac development and associated with a number of cardiac pathologies [1], served as an example here of a miRNA with an array of different sequence variants and demonstrated differential targeting properties of its two major 5′ isomiRs (Figure 3). Leading on from this, knowledge of miRNA variant expression will also improve the development of diagnostic tests of miRNA expression and allow greater precision in the design of miRNA mimics or anti-miRs as therapeutic agents.

In summary, our detailed compendium of cardiomyocyte miRNAs has revealed many unexpectedly abundant miR*, as well as unusual sequence variants and novel miRNA species. This raises interesting questions regarding their biological functions and specific modes of production that now await experimental characterization. It further highlights the fact that miRNA biogenesis and their impact on cellular processes is much more complex than originally anticipated. The diversity of miRNA sequences documented here will enrich our view of how these post-transcriptional regulators coordinate cardiomyocyte gene expression and more broadly, govern processes in cardiac biology and disease.

## Supporting Information

Text S1 **Supporting methods and results.**
(doc)

Figure S1 **Schematic of the sequence tag mapping approaches employed in this study.** Sequence tags were first mapped to hairpin sequences deposited in miRBase version 16 using the SOLiD™ Small RNA Pipeline. A custom script named the Mismatch and Multimapping Resolver (MMR) was then applied to deal with tags with mismatches and those that mapped to multiple miRNA loci (see Materials and Methods and Text S1 for details). Tags not mapping to miRBase were then mapped against the murine genome to mine for novel miRNAs.
(EPS)

Figure S2 **Global upregulation of miRNA expression in beating HL-1 cardiomyocytes.** (**A**) HL-1 cardiomyocytes progressed from a non-beating to a confluent, beating state over four days in two biological replicate experiments. RNA was harvested every 24 hours. (**B**) Normalized average miRNA expression levels on day 1 and 4 were compared on an M/A plot [71]. A miRNA expression threshold of >1000 tags per library was applied to increase stringency and only changes in expression exceeding two-fold in both replicates were considered significant (green dots). Nine miRNAs across a range of expression levels, which did not consistently show altered expression, were further selected as a reference set (dots in dark blue). (**C**) The set of reference miRNAs, as well as five that showed consistent down-regulation by SOLiD™ sequencing were independently quantified by qPCR across all four time points of HL-1 cell culture. Their expression level was further normalized to the average expression of 5 snoRNAs and to the average expression at all time points. Median expression of all miRNAs (denoted by a horizontal bar) on day one was then set to one. qPCR data are generated from one matched four-day time-course sample set. P-value was calculated using the Wilcoxon Matched-Pairs Signed-Ranks Test.
(EPS)

Figure S3 **Operational definition of miRNA features used in interpreting miRBase-mapped tags.** (**A**) All tags with a 5′ start position +/− 3 nt of a miRbase v16 annotated miRNA were counted as evidence of expression of that miRNA. These tags were considered as compatible with canonical Drosha and Dicer dependent cleavage and thus referred to as ‘generic’ miRNA tags. (**B**) Well-phased sets of tags mapping to the arm of a hairpin without a miRBase-annotated miRNA were classed as a novel miR*. A subset of these was classed as ‘generic’ as they are directly juxtaposed to a known miRNA. (**C**) Well-phased tag sets that mapped to hairpins outside the boundaries of miRBase annotated miRNAs were referred to as extreme isomiRs (e-miRs). To provide a unique identifier we have added the number and any other naming suffix from the known miRNA proximal to the new species. (**D**) Well-phased tag sets mapping to sequences antisense to known hairpin precursors and conforming to the criteria for novel miRNA discovery displayed in Figure S8 were taken as evidence for expression of antisense miRNAs (miR-as). We generally marked miRNAs that were already annotated in miRBase by open boxes, while filled boxes highlight novel species. The color convention used is blue for miRNAs on the 5′ arm, green on the 3′ arm. Discovery of novel miRNA species is exemplified in panels B and C for the 3′ arm of a hairpin; equivalent scenarios apply for the 5′ arm.
(EPS)

Figure S4 **Sequence mismatches within generic miRNA tags.** *Left,* proportion of miRBase-mapped tags without (grey) or with one or more mismatches at internal positions (blue) or at the 3′ end (red). *Top right*, Split of internal mismatches across all possible sequence changes. Note: an A-to-I editing event would manifest as an A-to-G substitution. *Bottom right*, 3′ terminal mismatches classed by non-templated base addition.
(EPS)

Figure S5 **Features of the long miR-301a.** (**A**) Predicted structure of miR-301a as deposited in miRbase v18, with most common isomiRs highlighted. Arrow shows asymmetrical loop, which may add extra nucleotides to the mature miR-301a. (**B**) miRNA Northern blot. Northern blots of HL-1 cell total RNA were probed for miR-301a, as well as miR-133a and miR-145 for reference (top panel). Length distribution of sequence tags for each miRNA in the HL-1 cell dataset is also shown (bottom panel). (**C**) miR-301a length distribution in other murine tissues. The proportion of miR-301a 3′ isomiRs of lengths 22–25 nt was determined by reanalysis of Illumina datasets deposited in miRbase v18 [22], [42]. Tissues with appreciable expression of miR-301a are shown along with our heart and HL-1 datasets.
(EPS)

Figure S6 **Nucleoside frequencies observed at hairpin positions surrounding the termini of cardiomyocyte miRNAs.** Nucleoside frequency at positions either side of the presumed processing sites was determined for each miRNA isomiR variant. For each miRNA, isomiR variant data were weighted according to their tag frequency; results were then given equal weight and averaged across all miRNAs per hairpin arm. For each sequence logo shown, the Y-axis denotes the frequency of that position being a specific nucleoside, with the size of the letter correlating to its frequency. Analysis was performed after separating miRNAs into (**A,B**) major/minor species derived from a given hairpin (≥80% or ≤20% of tags, respectively), for (**C**) miRNAs with symmetrical expression (tags >20% but <80%) and (**D**) for all miRNAs together. Background nucleotide frequency (top right) was calculated as a combination of all eight positions. Significant over-representation (#) and underrepresentation (*) are shown (p<0.01, two tailed t-test with Welch correction).
(EPS)

Figure S7 **Structural features of precursor hairpins surrounding miRNA termini.** The structural features of 229 precursor hairpins at miRNA termini were determined by visual inspection of the most common isomiR overlaid onto the predicted hairpins deposited in miRbase v18. The most common structures surrounding the presumed (**A**) Drosha and (**B**) Dicer cleavage sites are shown (cleavage positions based on 5′ start of miRNAs). The capital N^x^ denotes a nucleotide directly adjacent to a cleavage site. The lower case n^x^ denotes the opposing nucleotide. For each position only the major structural distortions are listed. For internal loops >2 nt the arrow indicates the direction the distortion extends. Analysis was performed on hairpins with at least one miRNA ≥150 tags and canonically processed. Analysis and display based on [37].
(EPS)

Figure S8 **Schematic of the novel miRNA discovery approaches employed in this study.** Genome-matched tags were size-selected and abundant well-phased tags set were identified. RNA secondary structures were predicted for surrounding genomic sequences and interrogated for a series of features characteristic of genuine pri-miRNA precursors (see Methods for a detailed description of this pipeline). Only novel miRNA candidates that complied with these criteria were shortlisted. The naming convention used was miR-N (for novel) followed by a serial number. Figure is 5′ arm focused, but novel miRNA occur on both arms of predicted hairpins.
(EPS)

Figure S9 **Validation of novel miRNAs by high stringency PCR and melt curve analysis.** PCR conditions were designed and optimised to specifically detect several novel miRNAs observed in the HL-1 dataset. RT-PCR was then performed on HL-1 and HeLa total RNA, with total RNA from *S.cerevisiae* and reactions containing HL-1 RNA but no reverse transcriptase acting as specificity controls. Amplification curves are shown to indicate relative abundance (though reactions are not considered strictly quantitative), melting curves shown to indicate specificity of the reaction (one peak indicates only one product is formed) and gel electrophoresis images are shown to demonstrate the single product is of the expected size.
(EPS)

Table S1 **Tag counts for all miRNAs and their features in the HL1 data set.**
(XLS)

Table S2 **Probes primers and mimic sequences.**
(DOC)

Table S3 **Known miRNA features detected in HL-1 cells.**
(DOC)

Table S4 **Most abundant generic miRNA tags in HL-1 cells.**
(DOC)

Table S5 **Examples of miRNAs with unexpected strand bias in HL-1 cardiomyocytes that have different strand bias in non-cardiac tissues‡.**
(DOC)

Table S6 Gene function analysis of the predicted targets of miRNAs with abundant miR* or biased miRNA-5p and -3p (>80% tags).
(DOC)

Table S7 **miRNAs with a high proportion of 5′ isomiRs in HL-1 cardiomyocytes (>20% of tags), which have low or variable 5′ isomiR levels in non-cardiac tissues ‡.**
(DOC)

Table S8 **Gene function analysis of the predicted targets of 5′ isomiR variants.**
(DOC)

Table S9 **miRNAs with internal sequence changes.**
(DOC)

Table S10 **miRNAs with >20% tags longer than 24 nt.**
(DOC)

Table S11 **Extreme isomiRs of known miRNAs.**
(DOC)

Dataset S1 **Alignment of tags derived from HL-1 cardiomyocytes with miRNA hairpins as listed in miRBase version 16.**
(HTML)

Dataset S2 **Alignment of tags derived from murine cardiac left ventricle with miRNA hairpins as listed in miRBase version 16.**
(HTML)

Dataset S3 **Novel miRNAs identified in HL-1 cardiomyocytes.**
(HTML)
