# Supplementary material for: Complexity of Murine Cardiomyocyte miRNA Biogenesis, Sequence Variant Expression and Function
Source: PLoS One. 2012 Feb 3;7(2):e30933. doi: 10.1371/journal.pone.0030933 (PMC3272019; doi:10.1371/journal.pone.0030933)
Supplement: Text S1 — Supporting methods and results. (doc) [file pone.0030933.s001.doc]

**Supporting Information**

**Supplementary Methods**

**Sequence analysis pipeline**

We refer to an individual deep sequence read as a tag and the number of times it occurred as a count. The SOLiD™ Small RNA Pipeline was used to map 18nt anchors of each tag to hairpin sequences obtained from miRBase, allowing a maximum of three mismatches and mapping to no more than fifteen loci before extending the alignment of the anchor. A cumulative maximum of 6 mismatches was tolerated in aligning tags to the reference plus adapter. A custom-made script, termed Mismatch and Multimapping resolver (MMR) applied an additional criteria where tags with isolated mismatches were only accepted if the position of the mismatch coincided with a quality value score <17. MMR examined miRBase mapped tags for genuine single base changes by requiring two adjacent colour space with quality values >17. Base changes at the last position prior to adapter sequence were flagged as non-templated additions while base changes at internal positions were considered potential edited sites. Tags representing multi-loci miRNAs were placed against the miRBase entry with the lowest entry number. For all other non-uniquely mapping tags a count was placed against each matching miRBase entry. miRNA expression levels were inferred from tag counts normalised to the total number of tags within each dataset and tags count ratios were calculate for each miRNA (day 4/day 1).

All tags that did not map to miRBase were mapped against the mouse genome (mm37 assembly), one chromosome at a time. To increase stringency, we used anchor length of 20nt, and allowed only 5 multiple mapping sites per chromosome with maximum two mismatches during anchor mapping and cumulative maximum of 6 mismatches. Tags were assigned to genomic locations with minimum number of mismatches and if tags mapped to more than one location with the same number of minimum mismatches they were assigned randomly. Next, 7nt genomic windows containing start positions for 150 or more tags (20-24nt long) were identified and overlapping 7nt windows were merged retaining a 7nt window with start positions for maximum tag count. After consolidation and optimization of overlapping windows, 6,369 candidate genomic regions encoding putative novel miRNAs (10,241,347 tags) and 108 loci potentially encoding antisense miRNAs (543,682 tags) passed this initial filter. A subsequent “authentic miRNA” filter was then applied by taking the 200nt flanking genomic sequence for each loci and bioinformatically predicting the secondary structures using RNALfold . Each structure had to form one stable hairpin (55-140nt) with adjusted minimum free energy (mfe) of <-20 kcal/mol (adjusted mfe = mfe / length of the structure). We also removed structures if either stem arm sequences or entire structure sequence contained <30% or >70% G/C nucleotide. To conform to known miRNA hairpin structures and requirements of a linear hairpin for Drosha/Dicer processing , we excluded structures with hairpin loops <3nt or >15nt, stem bulges >6nt, stem arms pairing less than 70% and structures with arms >20% different in length. We then required the predicted mature miRNA to be located within the stem of the predicted hairpin. The novel miRNA were then defined as 22nt in length with a 5’ start site determined by the most common start site of tags in the 7nt window. Structures were excluded if they overlapped with loci of known miRNAs. The structure with lowest adjusted minimum free energy for each remaining candidate novel miRNA was chosen and defined as the novel miRNA hairpin. Overlapping genomic features were identified using Ensembl PERL API v60.

A schematic of our tag mapping approach is shown in Figure S1 and details of tags mapped to miRBase are located in datasets S1, S2 and Table S1**.** Trimmed tags from a previously described dataset were mapped to miRbase version 16, allowing one mismatched base**.**

**Reanalysis of miR-301a expression in other tissues**

Next generation sequencing data deposited in miRBase version 17 and 18 was used to determine the relative proportion of miR-301a 3’ isomiR expression in several murine tissue types . Libraries corresponding to the same tissues and source were merged for analysis.

**Analysis of sequence and structural composition around miRNA processing sites**

For sequence analysis, all appreciably expressed generic miRNAs were considered. Nucleotide frequency at positions either side of the presumed processing sites was determined for each miRNA isomiR variant. For each miRNA, isomiR variant data were weighted according to their tag frequency; results were then given equal weight and averaged across all miRNAs per hairpin arm. Additionally, nucleotide frequencies were also calculated after separating miRNAs into major/minor species derived from a given hairpin (≥ 80% or ≤ 20% of tags, respectively) or miRNAs with symmetrical expression (tags >20% but <80%) and background nucleotide frequency was a combination of all analysed positions. A two tailed t-test with Welch correction was used to determine significant difference of nucleotide frequency from background.

For analysis of RNA structure around processing sites a set of appreciably expressed (at least one miRNA ≥ 150 tags) and canonically processed hairpins were visually inspected considering the most abundant miRNA isomiR variant only in each case and using predicted secondary structures as deposited in miRBase.

**Northern blotting**

Northern blotting was based on methods in . Specifically, 5µg of total HL-1 RNA was electrophoresed on a 10% polyacrylamide TBE-urea gel (Life Technologies) before being transferred onto GeneScreen Plus**®** membrane (PerkinElmer) in 0.5x TBE using a semi-dry blotting apparatus (Bio-Rad). Membranes were cross-linked (120 mJ) using a UV Stratalinker**®** 2400 (Stratagene) and pre-hybridised for 1 hour at 42°C in hybridisation buffer (7% SDS, 250mM sodium phosphate, pH 7.0). Oligonucleotides were ^32^P end-labelled with T4 polynucleotide kinase (NEB) according to the manufacturer’s instructions. Membranes were hybridised overnight at 42°C and washed 3 times in 2x SSC. Detection by phosphorimaging used an FLA-5100 imager and MULTIGAUGE software (Fujifilm). Probe sequences used were: miR-301a: GCT TTG ACA ATA CTA TTG CAC TG; miR-145: AAG GGA TTC CTG GGA AAA CTG GAC; miR-133: TAC AGC TGG TTG AAG GGG ACC AAA T.

**Reporter constructs and assays**

The two miRNA binding cassettes (*Ctfg*, and *Pdgf1*) were concatemers, AUUAA-Site 1-UGUCGACU-Site 2-UAUAAU-Site 3. The miR-133a isomiR site was derived from *Pgam1* (NM_023418.2; 1444-1473) and the canonical site was derived from *Ctgf* (NM_010217.2; 2322-2343).
The concatamer sites were inserted into the Not1 site behind the Renilla luciferase gene in the psiCHECK™-2 Vector (Promega) and verified by sequencing. MISSION® microRNA miR-133a Mimics (Sigma) were used: Can/22nt (UUUGGUCCCCUUCAACCAGCUG), Can/23nt (UUUGGUCCCCUUCAACCAGCUGU), iso/22nt (UUGGUCCCCUUCAACCAGCUGU), iso/23nt (UUGGUCCCCUUCAACCAGCUGUA) or control (UGUUAGCUGGAGUGAAAAC). In 24 well assays 30ng of reporter and MISSION® microRNA Mimics at a final concentration of 10nM (Sigma) were used in transient transfection assays as previously described .

**Supplementary Results**

**Global increase of miRNA expression as cardiomyocytes transition to a beating state**

We tested whether progression from a non-beating to a beating state altered miRNA expression (Figure S2A). We normalised expression for libraries from day one and day four of the HL-1 cell growth time course by dividing individual miRNA tag counts (all generic tags were treated as one entity) by the total number of miRBase-mapped tags and compared libraries by M/A plot (Figure S2B). Biological replicates were highly similar to each other, with Spearman rank correlation coefficients >0.9 (data not shown). To focus on high confidence candidates for differential expression, we required ≥2-fold change between day one and day four in both replicates and applied a threshold of 1000 tags per miRNA at each time-point (Figure S2B). miR-187, -92a, -20a, -99b*, -1196, -128-1, -211*, -504 and -702 then scored as down-regulated (green circles in Figure S2B; no miRNA showed consistent up-regulation). Interestingly, two of the down-regulated miRNAs are part of the oncogenic miR-17-92 cluster (miR-20a and miR-92) with demonstrated pro-proliferative functions . We established RT-qPCR protocols for four of these (miR-20a, -92a, -187, -702), and miR-7a (which fell below the 1000 tag threshold for one time-point), as well as nine apparently unregulated miRNAs of different abundance (miR-208a-5p, -133a, -208a-3p, -374, -29a, -145, -133a*, -542 and let-7a; dark blue circles in Figure S2B). After normalisation to the averaged expression of five snoRNAs (also obtained by qPCR), this revealed that the ‘unregulated’ miRNAs significantly increased in abundance from day one to day four (by 52%, p= 0.0039 Wilcoxon matched pair, signed-ranks test; Figure S2C). By contrast, levels of the five candidates tested for down-regulation decreased marginally (average of 33%). These results are consistent with the relative trends seen in the SOLiD™ data, given its inherent inability to detect bulk changes in miRNA level. Altogether, they indicate that, reminiscent of findings in other cell types , a global increase in levels is the most notable change in miRNA expression when HL-1 cardiomyocytes reach confluency.

**miRNA sequence changes in cardiomyocytes**

We looked for instances of internal A to G sequence change, which could be indicative of A to I editing by adenosine deaminases . Such changes were found in 0.35% of tags and similar in frequency to all other base substitutions (0.11 – 1.74% of tags; Figure S4). Applying an expression threshold of ≥150 tags per miRNA, and requiring at least 5% of all tags exhibiting an A to G change, we find only one example, miR-1274a, a tRNA-derived miRNA that was, however, removed from miRBase in the update from version 16 to version 17. To look for miRNA sequence polymorphisms or other undescribed editing phenomena, we applied the same thresholds to any other internal sequence change, which retained 32 miRNAs (Table S9). The most prevalent case was a G-to-A change at position 11 in 66% of tags mapping to miR-206, a miRNA that is required for proper cardiac muscle development . This sequence change is also observed in the heart biopsy sample. Though not in the “seed” region, this sequence change may still confer a selective function, since at least one of the targets of miR-206, *Hdac4*, is hypothesised to be bound by its centre region .

In summary, we find little evidence for editing by known mechanisms, consistent with reports that miRNA editing is rare even in brain tissue where RNA editing is thought to be more common .

miRNAs can have variable 3’ ends due to alternate Drosha and/or Dicer cleavage site preferences, as well as end trimming or non-templated 3’ additions (which may fortuitously match the pri-miRNA sequence or be unambiguous). We searched our dataset for instances of single nucleotide, unambiguous, non-templated addition and found these in 2.5% of our generic miRNA tags. miRNAs can be adenylated by GLD-2 or uridylated by Zcchc11 , however, we did not detect a general preference for uridilation or adenylation of miRNAs (Figure S4), nor did we observe individual miRNA species with common 3’ non-templated additions (>5% tags).

**Features of long cardiac miRNAs**

Although 90% of all tags were 24nt or shorter (Figure 1B), 28 miRNAs had >20% of tags that were longer than 24nt (Table S10), the same was found with 8 of 11 detectable cases in the lower coverage heart biopsy dataset. It has been shown that asymmetrical structural motifs present in precursor hairpins can lead to diversity in miRNA length , thus we hypothesized that longer miRNAs in HL-1 cells may be derived from such structures. We observed that 12/28 long miRNAs had asymmetrical structures that may have contributed to their length, with 7 of these strongly biased to one arm (resulting in a “short” miRNA on the opposing arm, average length 21nt). A further 5 miRNAs were characterized by Dicer or Drosha sites embedded in complex structures or large loops, 4 miRNAs had unstructured Drosha sites, 4 miRNAs had predicted structures inconsistent with canonical processing, 2 miRNAs were miR* of other long miRNA, leaving 1 miRNA with no obvious structural reason why it should be >24nt. 5 long miRNAs with asymmetrical structures also had Drosha/Dicer cleavage sites in complex structures and loop. Overall, this suggested that long miRNAs may arise from Dicer miscounting asymmetrically contributed nucleotides or greater inaccuracy of Dicer and Drosha cleavage in regions of low stability.

miR-301a is an extreme case of an abundant miRNA, with >60% of tags longer than 24nt in cardiomyocytes (Figure 2G) and a good example of an asymmetrical hairpin likely contributing extra nucleotides to the miRNA length (Figure S5A). The length of miR-301a was also confirmed by northern blotting (Figure S5B). Re-analysis of mouse datasets from other tissues showed that this miRNA is commonly >24nt and suggested its length is developmentally and tissue-specifically regulated (Figure S5C). Interestingly, both miR-301a and several members of the miR-30 family, which were also commonly longer than 24nt in our dataset (Table S10), target the mRNA for plasminogen activator inhibitor-1, a protein involved in the pathogenesis of cardiovascular disorders . Another prominent example was miR-181a, which is involved in cardiovascular development , yet 47% of tags were longer than 24nt in our cardiomyocyte dataset.

**Validation of novel miRNA species**

To validate the existence of our identified novel miRNAs, SYBR green-based qRT-PCR assays (based on the stem-loop extension assay described in ) were designed and optimised to detect the specific product. cDNA was made from the total RNA of HL-1 cells, HeLa cells and *S.cerevisiae* (where we would not expect to detect the miRNA). As a negative control HL-1 cell RNA was put into a reaction without reverse transcriptase. To determine specificity of detection, high temperature PCR reactions were performed on a Roche lightcycler™ for analysis of relative product abundance, product melting temperatures and then PCR products were run on a PAGE gel for accurate size discrimination (expected product sizes ranged from 56-60nt).

From HL-1 RNA, specific products were gained for miR-N4, miR-N29, miR-29* and miR-30e-as, while for miR-N4* there were some minor non-specific species of other sizes also amplified (Figure S9). miR-N4, miR-N4* and miR-30e-as were also detected in HeLa cells at lower abundance, suggesting these are expressed more widely. However miR-N29 and miR-N29* were restricted to HL-1 cells only. Some very low abundance non-specific products were seen in some reactions from the *S.cerevisiae* (miR-N4*, N4 and miR-29*) and –RT cDNA (miR-29*), which is common to all qPCR assays when their preferred template is not present (although in the case of miR-N4 this may be detecting a similar RNA in yeast).

In summary, by using high temperature qRT-PCR with melt curve and gel electrophoresis analysis, we have verified the expression of 5 novel miRNAs in HL-1 cardiomyocytes.

**Additional notes on miRbase 17 and 18 release**

During the preparation and review of this manuscript miRBase versions 17 and 18 were successively released. This added 135 new miRNAs and deleted 20 miRNAs. The relevant deleted miRNAs are referred to in the main text, one of the newly added miRNAs overlapped with our novel miRNA list (miR-5123) and two others overlapped with our novel miR* (miR-184 and miR-1196). Using our genome mapping data we determined if any of the other new miRbase entries were detectable ≥150 tags in our HL-1 dataset, even though they were not detected with our structural criteria for novel miRNAs. We were able to detect 9 new miRNAs at various expression levels (miR-5100, 3198 tags; -1843b, 1105; -5121, 901; -5099, 702; -3096b, 330; -378b, 194; -3968, 182; -5132, 157). Our relatively strict structural criteria would have eliminated 5 of these for lack of appropriate Drosha site structure, long terminal loops and/or low stability of the hairpin. The predicted structures for the remainder of the new miRBase entries were likely different from those deposited in miRBase and thus would also not meet our criteria. Overall, this comparison suggested that our novel miRNA discovery pipeline may have been overly conservative in some respects; nevertheless this tendency would also serve to reduce the false discovery rate.

**Supplementary References**

1. Hofacker IL, Priwitzer B, Stadler PF (2004) Prediction of locally stable RNA secondary structures for genome-wide surveys. Bioinformatics 20: 186-190.

2. Han J, Lee Y, Yeom KH, Nam JW, Heo I, et al. (2006) Molecular basis for the recognition of primary microRNAs by the Drosha-DGCR8 complex. Cell 125: 887-901.

3. Chiang HR, Schoenfeld LW, Ruby JG, Auyeung VC, Spies N, et al. (2010) Mammalian microRNAs: experimental evaluation of novel and previously annotated genes. Genes Dev 24: 992-1009.

4. Kuchen S, Resch W, Yamane A, Kuo N, Li Z, et al. (2010) Regulation of microRNA expression and abundance during lymphopoiesis. Immunity 32: 828-839.

5. Beilharz TH, Humphreys DT, Clancy JL, Thermann R, Martin DI, et al. (2009) microRNA-mediated messenger RNA deadenylation contributes to translational repression in mammalian cells. PLoS One 4: e6783.

6. Wang L, Feng Z, Wang X, Zhang X (2010) DEGseq: an R package for identifying differentially expressed genes from RNA-seq data. Bioinformatics 26: 136-138.

7. Olive V, Jiang I, He L (2010) mir-17-92, a cluster of miRNAs in the midst of the cancer network. Int J Biochem Cell Biol 42: 1348-1354.

8. Hwang HW, Wentzel EA, Mendell JT (2009) Cell-cell contact globally activates microRNA biogenesis. Proc Natl Acad Sci U S A 106: 7016-7021.

9. Kawahara Y, Zinshteyn B, Sethupathy P, Iizasa H, Hatzigeorgiou AG, et al. (2007) Redirection of silencing targets by adenosine-to-inosine editing of miRNAs. Science 315: 1137-1140.

10. Townley-Tilson WH, Callis TE, Wang D (2010) MicroRNAs 1, 133, and 206: critical factors of skeletal and cardiac muscle development, function, and disease. Int J Biochem Cell Biol 42: 1252-1255.

11. Winbanks CE, Wang B, Beyer C, Koh P, White L, et al. (2011) TGF-{beta} Regulates miR-206 and miR-29 to Control Myogenic Differentiation through Regulation of HDAC4. J Biol Chem 286: 13805-13814.

12. Katoh T, Sakaguchi Y, Miyauchi K, Suzuki T, Kashiwabara S, et al. (2009) Selective stabilization of mammalian microRNAs by 3' adenylation mediated by the cytoplasmic poly(A) polymerase GLD-2. Genes Dev 23: 433-438.

13. Jones MR, Quinton LJ, Blahna MT, Neilson JR, Fu S, et al. (2009) Zcchc11-dependent uridylation of microRNA directs cytokine expression. Nat Cell Biol 11: 1157-1163.

14. Starega-Roslan J, Krol J, Koscianska E, Kozlowski P, Szlachcic WJ, et al. (2011) Structural basis of microRNA length variety. Nucleic Acids Res 39: 257-268.

15. Patel N, Tahara SM, Malik P, Kalra VK (2011) Involvement of miR-30c and miR-301a in immediate induction of plasminogen activator inhibitor-1 by placental growth factor in human pulmonary endothelial cells. Biochem J 434: 473-482.

16. Huang ZP, Chen JF, Regan JN, Maguire CT, Tang RH, et al. (2010) Loss of microRNAs in neural crest leads to cardiovascular syndromes resembling human congenital heart defects. Arterioscler Thromb Vasc Biol 30: 2575-2586.

17. Chen C, Ridzon DA, Broomer AJ, Zhou Z, Lee DH, et al. (2005) Real-time quantification of microRNAs by stem-loop RT-PCR. Nucleic Acids Res 33: e179.

18. Clancy JL, Nousch M, Humphreys DT, Westman BJ, Beilharz TH, et al. (2007) Methods to analyze microRNA-mediated control of mRNA translation. Methods Enzymol 431: 83-111.

19. Matkovich SJ, Van Booven DJ, Eschenbacher WH, Dorn GW, 2nd (2011) RISC RNA Sequencing for Context-Specific Identification of In Vivo MicroRNA Targets. Circ Res 108: 18-26
