# Supplementary material for: Complexity of Murine Cardiomyocyte miRNA Biogenesis, Sequence Variant Expression and Function
Source: PLoS One. 2012 Feb 3;7(2):e30933. doi: 10.1371/journal.pone.0030933 (PMC3272019; doi:10.1371/journal.pone.0030933)
Supplement: Table S2 — Probes primers and mimic sequences. (DOC) [file pone.0030933.s012.doc]

**Table S2.** Probes primers and mimic sequences

|  | Full length RNA sequence | RT primer | Forward primer |
| --- | --- | --- | --- |
| snoRNA-55 | UGACUUAUAUAUCUGUCAAUCCCCUGAGA-GAUCACUGACGACUCCAUGUGUCUGAGCAA | GTCGTATCCAGTGCAGGGTCCGAGG-TATTCGCACTGGATACGACTTGCTC | For: CACTGACGACTCCATGTGTC  Common Rev: GTGCAGGGTCCGAGG |
| snoRNA-135 | CUAAAAUAGCUGGAAUUACCGGCAGAUUG-GUAGUGGUGAGCCUAUGGUUUUCUGAAG | GTCGTATCCAGTGCAGGGTCCGAGG-TATTCGCACTGGATACGACCTTCAG | TAGTGGTGAGCCTATGGTTTT |
| snoRNA-202 | GCUGUACUGACUUGAUGAAAGUACUUUU-GAACCCUUUUCCAUCUGAUG | GTCGTATCCAGTGCAGGGTCCGAGG-TATTCGCACTGGATACGACCATCAG | AGTACTTTTGAACCCTTTTCCA |
| snoRNA-234 | CUUUUGGAACUGAAUCUAAGUGAUUUAA-CAAAAAUUCGUCACUACCACUGAGA | GTCGTATCCAGTGCAGGGTCCGAGG-TATTCGCACTGGATACGACTCTCAG | TTAACAAAAATTCGTCACTACCA |
| snoRNA-412 | AUUAGCUGAAUGCUAACCUGAUGCAA-GUUACAAAUUACACUGAGA | GTCGTATCCAGTGCAGGGTCCGAGG-TATTCGCACTGGATACGACTCTCAG | CTAACCTGATGCAAGTTACAAATTAC |
| miR-let-7a | UGAGGUAGUAGGUUGUAUAGUU | GTCGTATCCAGTGCAGGGTCCGAGG-TATTCGCACTGGATACGACAACTAT | CCGCTGAGGTAGTAGGTTGT |
| miR-145 | GUCCAGUUUUCCCAGGAAUCCCU | GTCGTATCCAGTGCAGGGTCCGAGG-TATTCGCACTGGATACGACAGGGAT | CGTCCAGTTTTCCCAGGA |
| miR-133a | UUUUGGUCCCCUUCAACCAGCUG | GTCGTATCCAGTGCAGGGTCCGAGG-TATTCGCACTGGATACGACCAGCTG | TGTTTTGGTCCCCTTCAAC |
| miR-542 | CUCGGGGAUCAUCAUGUCACGA | GTCGTATCCAGTGCAGGGTCCGAGG-TATTCGCACTGGATACGACTCGTGA | GCTCGGGGATCATCATG |
| miR-374 | AUAUAAUACAACCUGCUAAGUG | GTCGTATCCAGTGCAGGGTCCGAGG-TATTCGCACTGGATACGACCACTTA | CGGCGATATAATACAACCTGC |
| miR-133a* | GCUGGUAAAAUGGAACCAAAU | GTCGTATCCAGTGCAGGGTCCGAGG-TATTCGCACTGGATACGACATTTGG | CGGCTGGTAAAATGGAA |
| miR-208a | UAUAAGACGAGCAAAAAGCUUGU | GTCGTATCCAGTGCAGGGTCCGAGG-TATTCGCACTGGATACGACACAAGC | GGCGTATAAGACGAGCAAAAA |
| miR-208a* | UGAGCUUUUGGCCCGGGUUAUAC | GTCGTATCCAGTGCAGGGTCCGAGG-TATTCGCACTGGATACGACGTATAA | TGAGCTTTTGGCCCGGG |
| miR-29a | UAGCACCAUCUGAAAUCGGUUA | GTCGTATCCAGTGCAGGGTCCGAGG-TATTCGCACTGGATACGACTAACCG | TGGCTAGCACCATCTGAAAT |
| miR-20a | UAAAGUGCUUAUAGUGCAGGUAG | GTCGTATCCAGTGCAGGGTCCGAGG-TATTCGCACTGGATACGACCTACCT | GGCCGTAAAGTGCTTATAGTGC |
| miR-702 | UGCCCACCCUUUACCCCGCUCC | GTCGTATCCAGTGCAGGGTCCGAGG-TATTCGCACTGGATACGACGGAGCG | TGCCCACCCTTTACCC |
| miR-187 | UCGUGUCUUGUGUUGCAGCCGG | GTCGTATCCAGTGCAGGGTCCGAGG-TATTCGCACTGGATACGACCCGGCT | GGTCGTGTCTTGTGTTGC |
| miR-92a | UAUUGCACUUGUCCCGGCCUGU | GTCGTATCCAGTGCAGGGTCCGAGG-TATTCGCACTGGATACGACACAGGC | CGTATTGCACTTGTCCCG |
| miR-7a | UGGAAGACUAGUGAUUUUGUUGUU | GTCGTATCCAGTGCAGGGTCCGAGG-TATTCGCACTGGATACGACAACAACA | GGCCTGGAAGACTAGTGATTT |
| miR-N4 | GGUAGCGUGGCCGAGCGGUCUA | GTCGTATCCAGTGCAGGGTCCGAGGT-  ATTCGCACTGGATACGACTAGACC | GGTAGCGTGGCCGAGC |
| miR-N4* | UCGAAUCCCACCGCUGCCAGGC | GTCGTATCCAGTGCAGGGTCCGAGGT-ATTCGCACTGGATACGACGCCTGG | TCGAATCCCACCGCTG |
| miR-N29 | CGGCGAUGAUGACACUCCAUA | GTCGTATCCAGTGCAGGGTCCGAGGT-ATTCGCACTGGATACGACTATGGA | GCGGCGATGATGACAC |
| miR-N29* | CCGGGUGAUGCGAAUCGUAAUCUG | GTCGTATCCAGTGCAGGGTCCGAGGT-ATTCGCACTGGATACGACCAGATT | CCGGGTGATGCGAATCGT |
| miR-30e-as | UGUAAACAUCCGACUGAAAGCU | GTCGTATCCAGTGCAGGGTCCGAGGT-ATTCGCACTGGATACGACAGCTTTC | TGCCTGTAAACATCCGACT |
