# Supplementary material for: Complexity of Murine Cardiomyocyte miRNA Biogenesis, Sequence Variant Expression and Function
Source: PLoS One. 2012 Feb 3;7(2):e30933. doi: 10.1371/journal.pone.0030933 (PMC3272019; doi:10.1371/journal.pone.0030933)
Supplement: Table S3 — Known miRNA features detected in HL-1 cells. (DOC) [file pone.0030933.s013.doc]

**Table S3.** Known miRNA features detected in HL-1 cells

| miRNA feature† | Expression threshold | |
| --- | --- | --- |
|  | None | ≥150  tags/miRNA |
| Mature | 431 | 192 |
| miRNA* | 382 | 119 |
| 5p | 115 | 40 |
| 3p | 107 | 45 |
| Total | 1035 | 403 |

† miRBase v16 lists 672 murine miRNA hairpin entries.
