# Supplementary material for: Complexity of Murine Cardiomyocyte miRNA Biogenesis, Sequence Variant Expression and Function
Source: PLoS One. 2012 Feb 3;7(2):e30933. doi: 10.1371/journal.pone.0030933 (PMC3272019; doi:10.1371/journal.pone.0030933)
Supplement: Table S4 — Most abundant generic miRNA tags in HL-1 cells. (DOC) [file pone.0030933.s014.doc]

**Table S4.** Most abundant generic miRNA tags in HL-1 cells

| miRNA | Fraction of total (%)† |
| --- | --- |
| miR-145 | 13.01 |
| miR-24 ‡ | 7.93 |
| miR-1 ‡ | 6.47 |
| miR-30d | 5.57 |
| miR-301a § | 3.89 |
| miR-29a | 3.30 |
| miR-30b | 3.04 |
| miR-125b-5p ‡ | 2.92 |
| miR-133a ‡ | 2.60 |
| miR-99a | 2.34 |
| miR-143 | 2.14 |
| miR-30c ‡ | 1.93 |
| miR-21 | 1.78 |
| miR-15b | 1.71 |
| miR-378 | 1.70 |
| miR-23b | 1.51 |
| miR-130a | 1.44 |
| miR-107 | 1.43 |
| miR-30a | 1.26 |
| miR-125a-5p | 1.25 |

† Calculated as a percentage of all miRBase-mapped tags. ‡ Multi-loci miRNAs. § This is the only miRNA hairpin in table *without* known function and/or expression in the heart as defined in and is expressed relatively lowly in the heart biopsy dataset (Supplementary Dataset S2).
