# Supplementary material for: Complexity of Murine Cardiomyocyte miRNA Biogenesis, Sequence Variant Expression and Function
Source: PLoS One. 2012 Feb 3;7(2):e30933. doi: 10.1371/journal.pone.0030933 (PMC3272019; doi:10.1371/journal.pone.0030933)
Supplement: Table S5 — Examples of miRNAs with unexpected strand bias in HL-1 cardiomyocytes that have different strand bias in non-cardiac tissues‡. (DOC) [file pone.0030933.s015.doc]

**Table S5. Examples of miRNAs with unexpected strand bias in HL-1 cardiomyocytes that have different strand bias in non-cardiac tissues‡**

| miRNA |  | miRNA expression (% of tags)† | | | | | | | | |
| --- | --- | --- | --- | --- | --- | --- | --- | --- | --- | --- |
|  | HL-1 | heart | e7.5‡ | e9.5‡ | e12.5‡ | newborn‡ | brain‡ | testes‡ | ovary‡ |  |
| **Symmetric expression of miR* in HL-1 cells (20-80% of tags) but low in any non-cardiac tissues (<20% of tags)** | | | | | | | | | |  |
| mmu-mir-322*§ | 58.43 | 44.46 | 6.70 | 0.51 | 3.86 | 4.16 | 5.11 | 3.91 | 5.23 |  |
| mmu-mir-22*§ | 57.60 | 37.59 | 0.35 | 0.43 | 0.61 | 0.32 | 1.33 | 0.91 | 0.30 |  |
| mmu-let-7d*§ | 30.51 | 28.28 | 0.07 | 0.10 | 0.06 | 0.37 | 0.24 | 0.92 | 1.21 |  |
| mmu-mir-361*§ | 20.23 | - | 5.64 | 7.37 | 3.32 | 6.90 | 9.48 | 15.87 | 9.92 |  |
| mmu-mir-872* | 57.47 | 49.25 | 13.67 | 8.82 | 13.00 | 10.83 | 15.39 | 7.07 | 19.64 |  |
| mmu-mir-503* | 22.64 | 47.03 | 2.78 | 5.16 | 1.75 | 9.93 | 12.09 | 7.10 | 2.41 |  |
| mmu-mir-425* | 28.34 | 31.89 | 3.55 | 2.55 | 3.27 | 4.64 | 5.79 | 1.55 | 3.93 |  |
| mmu-mir-7a-1* | 37.02 | 77.57 | 3.57 | 4.36 | 3.50 | 4.99 | 7.30 | 13.29 | 9.72 |  |
| mmu-mir-33* | 33.74 | 92.55 | 0.37 | 0.19 | 0.59 | 0.33 | 0.15 | - | - |  |
| mmu-mir-96* | 40.59 | - | 0.24 | 0.05 | 0.06 | 0.02 | - | - | - |  |
| mmu-mir-190* | 25.41 | 23.04 | - | - | 0.74 | 0.18 | 2.67 | - | - |  |
| mmu-mir-32* | 24.99 | 18.52 | 4.48 | 2.21 | 1.73 | 2.85 | 1.44 | 3.64 | - |  |
| mmu-mir-196b* | 49.46 | 66.67 | 1.23 | 0.74 | 0.91 | 0.66 | - | - | 2.57 |  |
| mmu-mir-9-1* | 25.63 | 9.05 | 6.38 | 2.17 | 10.81 | 2.97 | 5.45 | 3.45 | - |  |
| **Biased expression of 5p/3p miRNAs in HL-1 cells (>80% tags on one arm) but symmetric in all non-cardiac tissues (20-80% of tags per arm)** | | | | | | | | | |  |
| mmu-mir-151-5p§ | 87.14 | 84.12 | 27.71 | 56.62 | 30.25 | 48.58 | 33.35 | 26.40 | 40.78 |  |
| mmu-mir-423-3p§ | 93.42 | - | 38.53 | 52.4 | 26.96 | 46.86 | 56.12 | 60.51 | 34.89 |  |
| mmu-mir-3061-3p | 80.03 | 86.67 | 50.00 | 71.43 | 56.52 | 61.90 | 58.14 | - | - |  |
| mmu-mir-666-3p | 81.82 | 92.59 | 26.92 | 36.76 | 24.93 | 37.27 | 33.68 | 28.57 | 33.33 |  |
| mmu-mir-409-3p | 81.28 | 87.14 | 63.79 | 67.01 | 70.17 | 47.27 | 29.38 | 75.00 | 50.00 |  |
| **miR* displaying marked strand reversal in HL-1 cells (>80% of tags) but symmetric expression (20-80% of tags) or canonical expression (<20% of tags) in all non-cardiac tissues** | | | | | | | | | |  |
| mmu-mir-140*§ | 92.91 | 94.08 | 70.35 | 64.00 | 60.53 | 65.50 | 58.85 | 52.29 | 74.26 |  |
| mmu-mir-674* | 91.58 | 80.65 | 39.40 | 18.24 | 47.38 | 23.66 | 70.46 | 56.52 | 48.24 |  |
| mmu-mir-877* | 92.03 | 93.60 | 42.86 | 26.40 | 31.25 | 41.54 | 37.69 | 0.00 | 25.00 |  |
| mmu-mir-211*§ | 99.29 |  | 66.67 | - | 7.69 | 4.17 | - | - | - |  |
| mmu-mir-330*§ | 89.10 | 90.91 | 35.38 | 51.89 | 30.00 | 52.43 | 51.63 | 66.67 | 62.26 |  |
| mmu-mir-879* | 83.19 | - | 25.00 | - | 29.41 | 22.75 | 7.69 | - | - |  |
| mmu-mir-3074-1* | 89.12 | - | 0.20 | 0.03 | 0.52 | 0.07 | 0.24 | 0.33 | 0.04 |  |

† Calculated as a percentage of all miRBase-mapped tags. Heart is the left ventricle dataset “-“ represents <10 tags. ‡ Calculated from data sets described in . Only miRNAs were there was detectable expression in at least 3 tissues are included. § (pre-)miRNA with known function and/or expression in the heart as defined by .
