# Supplementary material for: Complexity of Murine Cardiomyocyte miRNA Biogenesis, Sequence Variant Expression and Function
Source: PLoS One. 2012 Feb 3;7(2):e30933. doi: 10.1371/journal.pone.0030933 (PMC3272019; doi:10.1371/journal.pone.0030933)
Supplement: Table S6 — Gene function analysis of the predicted targets of miRNAs with abundant miR* or biased miRNA-5p and -3p (>80% tags). (DOC) [file pone.0030933.s016.doc]

**Table S6**. Gene function analysis of the predicted targets of miRNAs with abundant miR* or biased miRNA-5p and -3p (>80% tags).

| miRNA and  Gene Function † | Targets/Total ‡ mature or 5p only | Targets/Total Common targets | | Targets/Total miR* or 3p only | | | |
| --- | --- | --- | --- | --- | --- | --- | --- |
| miR-211  Gene expression  Cardiovascular disease  Cardiovascular development and function | **58/322**  60/322  **28/322** | 1/11  2/11  1/11 | | miR* BIAS  **15/72**  **26/72**  **12/72** | | | |
|  |  |  | |  | | | |
| miR-140 |  |  | | miR* BIAS | | | |
|  |  |  | | miRBase | | | isomiR |
| Organ development  Cellular development | **25/127**  44/127 | <10 | | **28/128** | | | 29/190 |
|  |  |  |  | **42/128** | | | 45/190 |
|  |  |  | |  | | | |
| miR-330 |  |  | | miR* BIAS | | | |
|  |  | miRBase | isomiR | miRBase | isomiR | | |
| Gene expression | 45/141 | 5/16 | <10 | **88/354** | **107/401** | | |
| Tissue development | 14/141 | 3/16 |  | **77/354** | **92/401** | | |
|  |  |  | |  | | | |
| miR-877 |  |  | | miR* BIAS | | | |
| Cardiovascular disease  Gene expression | 1/33  9/33 | <10 | | miRBase | | isomiR | |
|  |  |  |  | 26/77 | | 30/155 | |
|  |  |  |  | 29/77 | | 29/115 | |
| miR-450b |  |  | | miRNA-3p bias | | | |
| Cardiovascular disease | **66/209** | <10 | | 32/101 | | | |
| Embryonic development | **33/209** |  | | **21/101** | | | |
| Tissue development | **39/209** |  | | **20/101** | | | |
|  |  |  | |  | | | |
| miR-22 |  |  | | miR* bias | | | |
| Cardiovascular system and function | 29/209 | <10 | | **17/146** | | | |
| Cardiovascular disease | **60/209** |  | | **42/146** | | | |
| Gene expression | **62/209** |  | | **54/146** | | | |
|  |  |  | |  | | | |
| miR-322 |  |  | | miR* bias | | | |
| Organ development | **80/646** | 1/34 | | **31/201** | | | |
| Gene expression | **153/646** | 11/34 | | **44/201** | | | |
|  |  |  | |  | | | |

† Definition of gene function by Ingenuity^TM^.

‡ Data shows number of target genes predicted by Targetscan with stated gene function/number of targets with any defined gene function. Bold denotes significant enrichment (p<0.01) of genes defined for said function, where grey denotes significant enrichment (0.01<p<0.05; Benjamini-Hochberg multiple testing correction method). Where a major 5’ isomiR exists data for the “miRBase” annotated and 5’ “isomiR” are shown separately.
