# Supplementary material for: Complexity of Murine Cardiomyocyte miRNA Biogenesis, Sequence Variant Expression and Function
Source: PLoS One. 2012 Feb 3;7(2):e30933. doi: 10.1371/journal.pone.0030933 (PMC3272019; doi:10.1371/journal.pone.0030933)
Supplement: Table S7 — miRNAs with a high proportion of 5′ isomiRs in HL-1 cardiomyocytes (>20% of tags), which have low or variable 5′ isomiR levels in non-cardiac tissues ‡. (DOC) [file pone.0030933.s017.doc]

**Table S7. miRNAs with a high proportion of 5’ isomiRs in HL-1 cardiomyocytes (>20% of tags), which have low or variable 5’ isomiR levels in non-cardiac tissues ‡**

| miRNA |  | 5’ IsomiR expression (% of tags)† | | | | | | | | |
| --- | --- | --- | --- | --- | --- | --- | --- | --- | --- | --- |
|  | HL-1 | heart | e7.5‡ | e9.5‡ | e12.5‡ | newborn‡ | brain‡ | testes‡ | ovary‡ |  |
| **Variable 5’ isomiR expression in non-cardiac tissues (≤20% of tags for at least one tissue)** | | | | | | | | | |  |
| mmu-mir-133b§ | 39.27 | 47.76 | 18.52 | - | 8.21 | 6.35 | 4.55 | 11.54 | 50.00 |  |
| mmu-mir-101b§ | 79.88 | 80.94 | 36.65 | 46.96 | 46.08 | 56.22 | 30.37 | 25.48 | 18.32 |  |
| mmu-mir-877* | 45.93 | - | - | 6.06 | - | 25.93 | 12.24 | - | - |  |
| mmu-mir-3096-5p | 70.72 | - | 5.00 | - | - | 30.00 | 56.25 | - | - |  |
| mmu-mir-1937b-1|| | 59.35 | 43.55 | 93.94 | - | 65.79 | 19.23 | 82.16 | 31.82 | 100.00 |  |
| mmu-mir-1983 | 87.97 | 17.55 | 19.75 | 42.86 | 14.91 | 40.46 | 9.36 | - | - |  |
| mmu-mir-450a-1* | 25.85 | - | 14.29 | 11.76 | 25.00 | 47.69 | - | - | - |  |
| mmu-mir-1944 | 72.16 | 18.80 | 11.56 | 20.18 | 12.50 | 32.32 | 7.47 | 29.17 | - |  |
| mmu-mir-1957 | 87.59 | 59.26 | 55.56 | 27.27 | 9.52 | 42.19 | - | - | - |  |
| mmu-mir-21*§ | 34.15 | - | 17.08 | 9.68 | 24.57 | 9.48 | 18.43 | 17.14 | 17.39 |  |
| mmu-mir-483§ | 93.67 | 16.67 | - | 52.63 | 34.78 | 61.49 | 3.13 | - | - |  |
| mmu-mir-339-3p | 26.06 | 50.00 | 35.20 | 4.30 | 34.15 | 13.72 | 57.79 | 32.00 | 38.46 |  |
| mmu-mir-222*§ | 80.60 | 20.00 | 76.19 | 93.02 | - | 90.00 | 12.77 | - | - |  |
| mmu-mir-670* | 80.96 | - | - | - | 28.00 | 4.60 | 2.13 | - | - |  |
| mmu-mir-199b§ | 30.32 | 59.46 | 11.06 | 35.71 | 56.23 | 24.10 | 18.34 | 30.23 | 7.58 |  |
| mmu-mir-677 | 67.86 | 35.29 | 64.29 | - | 23.53 | 16.67 | 20.00 | - | 0.00 |  |
| mmu-mir-34a* | 24.54 | 12.54 | 28.33 | - | 42.86 | 8.70 | 16.24 | 20.00 | - |  |
| mmu-mir-720 | 30.48 | 24.84 | 14.95 | 14.33 | 21.83 | 12.92 | 23.55 | 24.23 | 13.79 |  |
| mmu-mir-497*§ | 77.54 | - | 52.29 | 56.21 | 20.00 | 47.20 | 41.30 | - | 72.73 |  |
| mmu-mir-192 | 21.88 | 6.13 | 26.01 | 20.08 | 23.77 | 16.78 | 12.69 | 20.93 | 21.05 |  |
| mmu-mir-107*§ | 24.88 | - | - | 14.29 | - | 31.79 | 46.43 | - | - |  |
| mmu-mir-7a-1* | 20.03 | - | 11.36 | 14.29 | 9.95 | 55.00 | 8.81 | - | - |  |
| mmu-mir-342-5p§ | 38.72 | 85.00 | 15.56 | 25.89 | 18.14 | 15.87 | 24.24 | 18.18 | - |  |
| mmu-mir-125b-1*§ | 20.89 | - | 24.68 | - | 18.14 | 41.19 | 12.70 | - | 14.00 |  |
| mmu-mir-16-1*§ | 20.07 | 24.39 | 18.09 | 48.21 | 10.79 | 47.04 | 19.32 | - | - |  |
| mmu-mir-331-5p | 27.33 | 24.00 | 20.37 | 50.00 | 18.18 | 30.83 | 38.16 | - | - |  |
| mmu-mir-760-3p | 26.10 | 6.67 | - | - | 3.39 | 22.81 | 23.57 | - | - |  |
| mmu-mir-741 | 38.46 | - | - | 21.43 | - | 18.92 | - | 27.90 | - |  |
| **Low 5’ isomiR expression in all non-cardiac tissues (≤20% of tags)** | | | | | | | | | |  |
|  |  |  |  |  |  |  |  |  |  |  |
| mmu-mir-664* | 31.50 | 41.67 | 13.72 | 1.85 | - | 3.65 | 16.43 | - | - |  |
| mmu-mir-342-3p§ | 20.09 | 17.77 | 7.22 | 5.44 | 9.91 | 5.35 | 7.86 | 6.87 | - |  |
| mmu-mir-423-3p§ | 23.12 | 12.47 | 14.59 | 0.87 | 16.08 | 7.42 | 11.35 | - | 11.87 |  |
| mmu-mir-31*§ | 24.59 | 13.70 | - | 19.56 | - | 15.53 | 5.17 | - | - |  |
| mmu-mir-199a-1*§ | 30.32 | 12.55 | 13.71 | 17.58 | 11.08 | 18.17 | 16.66 | 6.30 | 10.88 |  |

† Calculated as a percentage of all miRBase-mapped tags. Heart is the left ventricle dataset. “-“ represents <10 tags ‡ Calculated from data sets described in . Only miRNAs were there was detectable expression in at least 3 tissues are included. § (pre-)miRNA with known function and/or expression in the heart as defined by . || miRBase v16 annotated miRNAs removed from miRBase v17.
