# Supplementary material for: Complexity of Murine Cardiomyocyte miRNA Biogenesis, Sequence Variant Expression and Function
Source: PLoS One. 2012 Feb 3;7(2):e30933. doi: 10.1371/journal.pone.0030933 (PMC3272019; doi:10.1371/journal.pone.0030933)
Supplement: Table S8 — Gene function analysis of the predicted targets of 5′ isomiR variants. (DOC) [file pone.0030933.s018.doc]

**Table S8.** Gene function analysis of the predicted targets of 5’ isomiR variants

| miRNA and  Gene Function † | Targets/Total ‡ Canonical only | Targets/Total Common | Targets/Total IsomiR only |
| --- | --- | --- | --- |
| miR-133a |  |  |  |
| Cardiovascular disease | **60/155** | 7/88 | 45/152 |
| miR-133a*  Cardiovascular disease | 1/58 | 1/21 | **68/189** |
|  |  |  |  |
| miR-100* |  |  |  |
| Cardiovascular development and function | **14/113** | 0/11 | 8/65 |
| Organ development | **24/113** | 2/11 | 14/65 |
| miR-140* |  |  |  |
| Cardiovascular development and function | 19/134 | 4/28 | **26/196** |
| Organ development | **31/134** | 9/28 | **32/196** |
| Cellular development  Gene expression | **45/134**  **43/134** | 9/28  10/28 | **37/196**  5/196 |
|  |  |  |  |
| Let-7g* |  |  |  |
| Gene expression | **110/371** | 34/125 | **78/276** |
| Cardiovascular disease  Cardiovascular development and function | **106/371**  **43/371** | 37/125  7/125 | 71/276  5/276 |
|  |  |  |  |
| miR-222  Cardiovascular disease  Tissue development | 20/56  11/56 | **31/97**  18/97 | 5/77  16/77 |
|  |  |  |  |
| miR-1957  Tissue development  Cell-cell signalling and adhesion | 18/80  15/80 | 1/3  0/3 | **44/162**  **36/162** |
|  |  |  |  |
| miR-101a  Cardiovascular disease  Tissue development  Gene expression | **88/296**  39/296  65/296 | 53/185  **46/185**  **53/185** | 29/88  **23/88**  **31/88** |

† Definition of gene function by Ingenuity^TM^.

‡ Data shows number of target genes predicted by Targetscan with stated gene function/number of targets with any defined gene function. Bold denotes significant enrichment (p<0.01) of genes defined for said function, where grey denotes significant enrichment (0.01<p<0.05; Benjamini-Hochberg multiple testing correction method).
