# Supplementary material for: Complexity of Murine Cardiomyocyte miRNA Biogenesis, Sequence Variant Expression and Function
Source: PLoS One. 2012 Feb 3;7(2):e30933. doi: 10.1371/journal.pone.0030933 (PMC3272019; doi:10.1371/journal.pone.0030933)
Supplement: Table S9 — miRNAs with internal sequence changes. (DOC) [file pone.0030933.s019.doc]

**Table S9.** miRNAs with internal sequence changes.

| miRNA | Base Change † | % of tags | Counts | Seed region affected and other comments ‡ |
| --- | --- | --- | --- | --- |
| mir-130a | GA | 25.99 | 150641 | No |
| mir-125b-1 | TA | 10.57 | 126149 | No |
| miR-29a | GA | 6.72 | 90300 | No |
| mir-125b-1 | TC | 6.88 | 82079 | No |
| mir-206 | GA | 66.54 | 40500 | No |
| miR-322 | TA | 10.07 | 18184 | No |
| mir-1939 | TG | 41.35 | 13327 | No – removed from miRBase v17 |
| miR-133a-1 | CT | 12.46 | 12995 | No |
| mmu-mir-1274a | AI | 48.06 | 12951 | No – extreme isomiR - removed from miRBase v17 |
| miR-125a | CT | 10.66 | 11114 | No |
| miR-29c | GA | 7.81 | 10399 | No |
| miR-532 | GA | 12.70 | 7299 | No |
| miR-320 | GA | 7.75 | 3897 | No |
| mir-450a-1 | TC | 11.02 | 3655 | No |
| miR-15b* | GA | 6.22 | 2607 | No |
| miR-720 | CA | 13.71 | 2094 | No |
| miR-93* | TA | 7.82 | 1693 | No |
| miR-362* | GA | 5.14 | 1523 | No |
| miR-222 | GA | 13.36 | 1510 | No |
| miR-421 | GA | 18.53 | 1504 | No |
| miR-1-1* | GA | 5.14 | 1202 | No |
| miR-138-1 | GA | 8.85 | 1052 | No |
| miR-1196 | GA | 5.42 | 1014 | No |
| miR-324* | GA | 8.39 | 740 | No |
| mir-181d | TC | 31.01 | 662 | No |
| miR-1937c | AC | 26.42 | 575 | No – removed from miRBase v17 |
| miR-28* | GA | 9.95 | 525 | No |
| mmu-mir-1983 | GT | 9.74 | 490 | No - extreme isomiR |
| miR-195* | GA | 5.65 | 294 | No |
| miR-106a | AT | 7.96 | 281 | No |
| miR-130b | GA | 10.42 | 248 | No |
| miR-99a* | GA | 10.03 | 219 | No |
| mmu-mir-344g | TA | 41.55 | 150 | No- extreme isomiR |

† Exact positions are shown in Supplementary data sets S1 and S2 .

‡ Extreme isomiRs as described in Table 3.
