# Supplementary material for: Complexity of Murine Cardiomyocyte miRNA Biogenesis, Sequence Variant Expression and Function
Source: PLoS One. 2012 Feb 3;7(2):e30933. doi: 10.1371/journal.pone.0030933 (PMC3272019; doi:10.1371/journal.pone.0030933)
Supplement: Table S10 — miRNAs with >20% tags longer than 24 nt. (DOC) [file pone.0030933.s020.doc]

**Table S10.** miRNAs with >20% tags longer than 24nt.

| miRNA | % miRNA>24nt | Counts > 24nt |
| --- | --- | --- |
| mmu-mir-301a | 61.85 | 977581 |
| mmu-mir-351 | 67.6 | 140599 |
| mmu-mir-30e | 26.79 | 130725 |
| mmu-mir-182 | 55.15 | 117928 |
| mmu-mir-181a-1 | 46.95 | 92030 |
| mmu-mir-3102 | 46.02 | 70848 |
| mmu-mir-181b-1 | 36.02 | 46294 |
| mmu-mir-342* | 27.86 | 21643 |
| mmu-mir-301b | 59.35 | 19859 |
| mmu-mir-362 | 39.17 | 13584 |
| mmu-mir-702 | 69.27 | 9421 |
| mmu-mir-501 | 40.13 | 8790 |
| mmu-mir-671 | 66.88 | 6150 |
| mmu-mir-99b* | 26.44 | 5724 |
| mmu-mir-361* | 22.69 | 4340 |
| mmu-mir-29a* | 53.97 | 3979 |
| mmu-mir-222 | 34.06 | 3850 |
| mmu-mir-3068 | 49.01 | 2801 |
| mmu-mir-3096 | 50.58 | 2683 |
| mmu-mir-1944 | 73.04 | 2238 |
| mmu-miR-330* | 34.29 | 2155 |
| mmu-mir-342 | 67 | 1892 |
| mmu-mir-181d | 78.41 | 1674 |
| mmu-mir-27b* | 33.51 | 1217 |
| mmu-mir-500* | 43.54 | 687 |
| mmu-mir-877 | 21.77 | 459 |
| mmu-mir-212 | 30.22 | 366 |
| mmu-mir-330 | 20.95 | 141 |
