# Supplementary material for: Complexity of Murine Cardiomyocyte miRNA Biogenesis, Sequence Variant Expression and Function
Source: PLoS One. 2012 Feb 3;7(2):e30933. doi: 10.1371/journal.pone.0030933 (PMC3272019; doi:10.1371/journal.pone.0030933)
Supplement: Table S11 — Extreme isomiRs of known miRNAs. (DOC) [file pone.0030933.s021.doc]

**Table S11.** Extreme isomiRs of known miRNAs.

| **miRNA** | **% of Hairpin** † | **Counts** | **Type** ‡ | **Notes** |
| --- | --- | --- | --- | --- |
| e-miR-721 | 99.7 | 76794 | -9 isomiR | Stem into loop |
| e-miR-3072 | 99.97 | 43015 | -6 isomiR |  |
| e-miR-1274a | 96.06 | 26950 | -4 isomiR | A to I editing; Removed from miRBase 17 (homologous to tRNA) |
| e-miR-1983 | 54.22 | 5029 | -6 isomiR |  |
| e-miR-1937b-1 | 46.77 | 3718 | -6 isomiR |  |
| e-miR-1947 | 97.55 | 2587 | -9 isomiR | Loop into stem |
| e-miR-1933-5p | 77.40 | 1267 | -7 isomiR | 18-19 nt |
| e-miR-136 | 98.31 | 1162 | +4 isomiR |  |
| e-miR-690 | 92.78 | 938 | -21 isomiR | Loop into stem |
| e-miR-711 | 98.8 | 925 | +8 isomiR | Stem into loop |
| e-miR-3473 | 45.87 | 921 | -4 isomiR |  |
| e-miR-463 | 48.4 | 757 | -8 isomiR | Loop into stem |
| e-miR-697 | 97.08 | 498 | -12 isomiR | Loop into stem |
| e-miR-592 | 92.13 | 492 | +14 isomiR | Stem into loop |
| e-miR-344g-5p | 97.08 |  | +20 isomiR | Stem into loop |
| e-miR-2137 | 30.87 | 321 | -4 isomiR | Loop into stem |
| e-miR-144 | 89.86 | 319 | -17 isomiR | 5p stem – loop – 3p stem |
| e-miR-743b-5p | 23.75 | 242 | -6 isomiR |  |
| e-miR-715 | 69.35 | 212 | +19 isomiR | Stem into loop; Removed from miRBase 17 (homologous to tRNA) |
| e-miR-370 | 97.41 | 188 | +9 isomiR |  |
| e-miR-3067 | 100 | 176 | +13 isomiR | Stem into loop |
| e-miR-448-5p | 100 | 175 | +11 isomiR |  |
| e-miR-669o-5p | 99.35 | 152 | +20 isomiR |  |
| e-miR-1934* | 73.05 | 1173 | +11 isomiR |  |
| e-miR-194-1* | 54.74 | 497 | -7 isomiR | Loop into stem |
| e-miR-455* | 68.65 | 335 | -15 isomiR |  |
| e-miR-881* | 56.21 | 190 | -5 isomiR |  |
| e-miR-669e | 97.9 | 1432 | REMOVED | Repetitive sequence |
| e-miR-1935 | 54.2 | 798 | REMOVED | Lack of well phased tag set |
| e-miR-706 | 84.5 | 754 | REMOVED | Lack of well phased tag set |
| e-miR-1990 | 98 | 686 | REMOVED | Lack of well phased tag set |
| e-miR-1954 | 96.2 | 451 | REMOVED | Lack of well phased tag set |
| e-miR-1946b | 33.8 | 420 | REMOVED | Elevated noise |
| e-miR-1192 | 76.6 | 418 | REMOVED | Lack of well phased tag set |
| e-miR-669h | 98.6 | 356 | REMOVED | Repetitive sequence |
| e-miR-615 | 44.5 | 300 | REMOVED | Elevated noise |
| e-miR-1946a | 34.4 | 296 | REMOVED | Elevated noise |
| e-miR-759 | 93.7 | 268 | REMOVED | Lack of well phased tag set |
| e-miR-3470a | 49.7 | 266 | REMOVED | Lack of well phased tag set |
| e-miR-1186 | 43.2 | 261 | REMOVED | Elevated noise |
| e-miR-466k | 80.3 | 248 | REMOVED | Lack of well phased tag set |
| e-miR-433 | 99.2 | 239 | REMOVED | Continued 5’ isomiR |
| e-miR-335 | 43.7 | 224 | REMOVED | Lack of well phased tag set |
| e-miR-466i | 73.9 | 212 | REMOVED | Repetitive sequence |
| e-miR-1966 | 88.6 | 195 | REMOVED | Lack of well phased tag set |
| e-miR-1895 | 81.5 | 189 | REMOVED | Repetitive sequence |
| e-miR-683 | 90.1 | 173 | REMOVED | Lack of well phased tag set |
| e-miR-1904 | 93.0 | 172 | REMOVED | Lack of well phased tag set |
| e-miR-466f | 64.8 | 171 | REMOVED | Repetitive sequence |
| e-miR-135b | 69.9 | 158 | REMOVED | Elevated noise |
| e-miR-669g | 72.2 | 156 | REMOVED | Elevated noise |
| e-miR-1905 | 89.3 | 150 | REMOVED | Lack of well phased tag set |

† Percentage contribution of e-isomiR to total tags mapped to the hairpin. ‡ REMOVED means that there was not sufficient evidence that the observed tags represented a *bona fide* miRNA (reason for removal given in comments).
