# Supplementary material for: Complexity of Murine Cardiomyocyte miRNA Biogenesis, Sequence Variant Expression and Function
Source: PLoS One. 2012 Feb 3;7(2):e30933. doi: 10.1371/journal.pone.0030933 (PMC3272019; doi:10.1371/journal.pone.0030933)
Supplement: Dataset S1 — Alignment of tags derived from HL-1 cardiomyocytes with miRNA hairpins as listed in miRBase version 16. (HTML) [file pone.0030933.s022.html]

  
  
Humphreys et al
  
  
Dataset S1: Alignment of tags# derived from HL-1 cardiomyocytes with miRNA hairpins as listed in miRBase version 16  
  
# Underlined bases denote a base change. The miR\* sequence is the miRBase annotated miR\* or the position of a putative generic miR\* where one is not annotated in miRBase. Coloured bases at the 3� end denote different observed 3� end positions and tag count of these variants is shown in same colour to the right. miRBase listed secondary structure of miRNA hairpins is also shown.
  
  

  
  
If this is the only text that loads, enable javascript in your browser.
