# Supplementary material for: Complexity of Murine Cardiomyocyte miRNA Biogenesis, Sequence Variant Expression and Function
Source: PLoS One. 2012 Feb 3;7(2):e30933. doi: 10.1371/journal.pone.0030933 (PMC3272019; doi:10.1371/journal.pone.0030933)
Supplement: Dataset S3 — Novel miRNAs identified in HL-1 cardiomyocytes. (HTML) [file pone.0030933.s024.html]

  
  
Humphreys et al  
Dataset S3 Novel miRNAs identified in HL-1 cardiomyocytes.
  
  
#Position of predicted miRNA precursor \* GO terms derived from Ingenuity gene function analysis. Bold denotes novel miRNAs with a detectable miR\*.
  
  

  
  
If this is the only text that loads, enable javascript in your browser.
